# Human Liver Stem Cell-Derived Extracellular Vesicles Prevent Aristolochic Acid-Induced Kidney Fibrosis

**DOI:** 10.3389/fimmu.2018.01639

**Published:** 2018-07-19

**Authors:** Sharad Kholia, Maria Beatriz Herrera Sanchez, Massimo Cedrino, Elli Papadimitriou, Marta Tapparo, Maria Chiara Deregibus, Maria Felice Brizzi, Ciro Tetta, Giovanni Camussi

**Affiliations:** ^1^Department of Medical Sciences, University of Torino, Torino, Italy; ^2^Molecular Biotechnology Centre, University of Torino, Torino, Italy; ^3^2i3T Società per la gestione dell’incubatore di imprese e per il trasferimento tecnologico Scarl, University of Torino, Torino, Italy; ^4^Department of Molecular Biotechnology and Health Science, University of Torino, Torino, Italy; ^5^Unicyte AG, Oberdorf, Switzerland

**Keywords:** aristolochic acid nephropathy, extracellular vesicles, human liver stem cells, chronic kidney disease, miRNA, fibrosis

## Abstract

With limited therapeutic intervention in preventing the progression to end-stage renal disease, chronic kidney disease (CKD) remains a global health-care burden. Aristolochic acid (AA) induced nephropathy is a model of CKD characterised by inflammation, tubular injury, and interstitial fibrosis. Human liver stem cell-derived extracellular vesicles (HLSC-EVs) have been reported to exhibit therapeutic properties in various disease models including acute kidney injury. In the present study, we aimed to investigate the effects of HLSC-EVs on tubular regeneration and interstitial fibrosis in an AA-induced mouse model of CKD. NSG mice were injected with HLSC-EVs 3 days after administering AA on a weekly basis for 4 weeks. Mice injected with AA significantly lost weight over the 4-week period. Deterioration in kidney function was also observed. Histology was performed to evaluate tubular necrosis, interstitial fibrosis, as well as infiltration of inflammatory cells/fibroblasts. Kidneys were also subjected to gene array analyses to evaluate regulation of microRNAs (miRNAs) and pro-fibrotic genes. The effect of HLSC-EVs was also tested *in vitro* to assess pro-fibrotic gene regulation in fibroblasts cocultured with AA pretreated tubular epithelial cells. Histological analyses showed that treatment with HLSC-EVs significantly reduced tubular necrosis, interstitial fibrosis, infiltration of CD45 cells and fibroblasts, which were all elevated during AA induced injury. At a molecular level, HLSC-EVs significantly inhibited the upregulation of the pro-fibrotic genes α*-Sma, Tgfb1*, and *Col1a1 in vivo* and *in vitro*. Fibrosis gene array analyses revealed an upregulation of 35 pro-fibrotic genes in AA injured mice. Treatment with HLSC-EVs downregulated 14 pro-fibrotic genes in total, out of which, 5 were upregulated in mice injured with AA. Analyses of the total mouse miRnome identified several miRNAs involved in the regulation of fibrotic pathways, which were found to be modulated post-treatment with HLSC-EVs. These results indicate that HLSC-EVs play a regenerative role in CKD possibly through the regulation of genes and miRNAs that are activated during the progression of the disease.

## Introduction

Chronic kidney disease (CKD) is a pathology that affects billions of individuals globally (1). Currently, treatment for end-stage CKD is limited to haemodialysis and kidney transplantation, both of which are restricted to financial restraint and/or availability of donor kidneys (2). Interstitial fibrosis, a hallmark of CKD is now accepted as an independent contributor/predictor of disease progression, therefore, making it a potential target for therapy (3). Fibrosis is mainly characterised by the release of inflammatory mediators, infiltration of inflammatory/immune cells, activation and proliferation of resident fibroblasts, epithelial to mesenchymal transition (EMT), and dysregulation of extracellular matrix (ECM) deposition (4). Aristolochic acid nephropathy (AAN) is a rapidly progressing form of kidney disease that occurs in patients who have ingested plants containing aristolochic acid (AA) as part of traditional herbal therapy (Chinese herb nephropathy) or as a food contaminant (Balkan endemic nephropathy) (5). AAN is predominantly characterised by interstitial fibrosis accompanied with tubular atrophy and EMT that eventually progresses towards end-stage renal disease (5). There is also a potential of developing urothelial malignancies due to DNA adducting properties of AA (5). Although, various pathways have been implicated in kidney fibrosis, the transforming growth factor beta (TGFβ) signalling pathway remains to be a major contributor (4).

Stem cell-based therapies have gathered a substantial amount of interest over the past decade. They have been implicated as potential treatments in various disease conditions including acute and chronic renal injury. For instance, mesenchymal stem cells (MSC) were shown to exhibit a protective role in an animal model of obstruction-induced renal fibrosis by reducing the activation of signal transducer and activator of transcription 3 (6). Furthermore, human amniotic fluid derived-stem cells played a protective role by alleviating interstitial fibrosis in a mouse model of unilateral ureter obstruction (2). Human liver stem cells (HLSCs), a stem cell population resident in the liver, discovered in our lab (7), also exhibit mesenchymal and embryonic markers together with a multipotency potential to differentiate into several types of cells (7). Over the years, we have found these cells to display remarkable therapeutic properties. In a model of acetaminophen-induced liver injury, HLSCs proved to contribute towards the regeneration of liver parenchyma (7). Whereas, in a model of fulminant liver failure, mouse survival rate was increased after treatment with HLSCs (8). Nonetheless, the regenerative potential of HLSCs is not limited to the liver only. In a model of acute kidney injury (AKI), mice treated with HLSCs showed improved renal morphology and function substantially (9).

The mechanism through which stem cells exert their biological effect has been suggested to be of paracrine in nature. Factors released by stem cells, either directly or through packaged vesicles, interact with recipient cells and exert therapeutic effects (10). In fact, the mechanism and function through which cells package and transfer biologically active molecules in the form of extracellular vesicles (EVs) has gained huge interest over the last few years (10). EVs are heterogeneous lipid membrane vesicles released by cells either through membrane shedding or fusion of multivesicular bodies with the cell membrane (11). They tend to influence target cells by delivering biologically active cargo such as: proteins, lipids, and nucleic acids (11).

Therapeutic effects exhibited by stem cell-derived EVs are very well documented in the scientific literature. For instance, Bruno et al. (12) showed that MSC-derived EVs not only increased survival but also promoted overall healing and proliferation of tubular epithelial cells in a mouse model of AKI. In a model of renal ischaemia/reperfusion, MSC-derived EVs reduced injury and augmented renal recovery compared to untreated mice (13). HLSC-derived EVs, like MSC-EVs, have also been functionally and molecularly characterised (14). In addition, like their cellular counterpart, human liver stem cell-derived extracellular vesicles (HLSC-EVs) also exhibit regenerative properties in various models of tissue pathology including AKI (9, 15).

The therapeutic potential of EVs derived from HLSCs in CKD and, in particular, AAN has yet to be elucidated. The aim of this study was, therefore, to investigate whether EVs from HLSCs contribute towards tubular regeneration and alleviate fibrosis in a chronic mouse model of AAN.

## Materials and Methods

### Isolation and Characterisation of EVs

Extracellular vesicles were obtained from supernatants of HLSCs (2 × 10^6^ cells/T75 flask) or MRC5 human dermal fibroblasts cultured in serum-free Roswell Park Memorial Institute Medium (RPMI) (Euroclone S.p.A, Italy) for 18 h. Viability of cells at the time of supernatant collection was 98% as confirmed by Trypan blue exclusion. Briefly, supernatants were centrifuged at 3,000 *g* for 15 min at 4°C for the removal of cell debris and apoptotic bodies, followed by ultracentrifugation at 100,000 *g* for 2 h at 4°C (Beckman Coulter Optima L-90 K, Fullerton, CA, USA). The pellet of EVs obtained was resuspended in RPMI supplemented with 1% dimethyl sulfoxide (DMSO) and stored at −80°C until use. Further purification of EVs was performed by iodixanol (Optiprep, Sigma, St. Louis, MO, USA) floating density separation protocol as described previously (16). The protocol was modified from the initial one described by Kowal et al. (17) to accommodate for larger centrifugation volumes to obtain sufficient amounts of EVs for *in vivo* experiments. Briefly, EVs acquired through ultracentrifugation were resuspended in 500 µl of 60% iodixanol supplemented with 0.25 M sucrose. One ml of 30, 15, and 5% iodixanol working solution was layered sequentially above the EV/60% iodixanol suspension and the final volume adjusted to 10 ml with saline solution. The tubes were ultracentrifuged at 350,000 *g* for 1 h at 4°C without brake in an Optima L-100K ultracentrifuge (Beckman Coulter) equipped with Type 90Ti rotor. The 15, 30, and 60% fractions were recovered, diluted in PBS and re-ultracentrifuged at 100,000 *g* for 1 h at 4°C. The pellet obtained was resuspended in PBS/1% DMSO for subsequent studies. EVs were mainly detected in the 15% fraction as determined by the Nanosight LM10 system (NanoSight, Wiltshire, UK) (Figure S1A in Supplementary Material). EVs isolated from the 15% fraction were used for experiments.

Characterisation of EVs was performed by cytofluorimetric analyses. HLSC-EVs were positive for the typical mesenchymal surface markers characteristic of HLSCs such as CD29, CD44, CD73, and CD90 as well as the exosomal markers CD81 and CD107 as described before (9). A further characterisation was performed by electron microscopy showing the presence of vesicles ranging between 40 and 100 nm (15) (Figure S1B in Supplementary Material). Western blot analyses of EV protein also confirmed the presence of classical exosomal markers such as CD63, CD81, and TSG101 as described previously (9) (Figure S1C in Supplementary Material).

For EV internalisation experiments, EVs were labelled with 1 µM Dil dye (Thermo Fisher Scientific, Waltham, MA, USA) as described before (15). Briefly, purified EVs were resuspended in PBS together with 1 µM Dil dye and ultracentrifuged for 1 h at 4°C. The pellet of EVs obtained was washed once by ultracentrifugation and resuspended in PBS/1% DMSO for use in experiments.

Quantification and size distribution of purified EVs was determined by Nanosight (NanoSight, Wiltshire, UK). Briefly, EV preparations were diluted (1:200) in sterile saline solution and analysed by the Nanoparticle Analyses System using the NTA 1.4 Analytical Software as described previously (9).

### Cell Culture

#### Human Liver Stem Cell

Human liver stem cells were isolated from human cryopreserved normal adult hepatocytes (Lonza, Basel, Switzerland) as described before (9). Briefly, hepatocytes were cultured in Hepatozyme-SFM medium (Lonza, Basel, Switzerland) for 2 weeks to allow majority of the hepatocytes to die. The surviving population of cells were cultured in alpha minimum essential medium/endothelial basal medium-1 (3:1) (Lonza, Basel, Switzerland) supplemented with l-glutamine (5 mM), HEPES (12 mM, pH 7.4), penicillin (50 IU/ml), streptomycin (50 µg/ml) (all from Sigma, St. Louis, MO, USA), and 10% fetal calf serum (FCS) (Invitrogen, Carlsbad, CA, USA). Cells were expanded, characterised, and cryo-preserved as described previously (9).

Human liver stem cells were positive for the mesenchymal stem cell markers, but not haematopoietic and endothelial markers as described before (9). In addition, they were positive for human albumin, alpha-fetoprotein, resident stem cell markers such as vimentin and nestin, and negative for CD34, CD117, and cytokeratin 19 oval cell markers as reported previously (9). Embryonic stem cell markers such as Nanog, Oct4, Sox2, and SSEA4 were also positively expressed in HLSCs (9). Stemness of HLSCs was confirmed by endothelial, osteogenic, and hepatic differentiation under appropriate culture conditions as described earlier (9).

#### MRC5 Human Lung Fibroblasts

The human fetal lung fibroblast like cell line MRC5 PD 19 (purchased from Sigma, St. Louis, MO, USA) was adopted for the isolation of EVs as a negative control for *in vivo* studies. Briefly, cells were cultured in Dulbecco’s modified essential medium (DMEM) low glucose supplemented with l-glutamine (5 mM), HEPES (12 mM, pH 7.4), penicillin (50 IU/ml), streptomycin (50 µg/ml), and 10% FCS. At a confluency of 90%, cells were starved overnight in serum-free RPMI. The supernatant was collected and subjected to EV isolation as mentioned above.

#### Mouse Tubular Epithelial Cells (mTEC)

Mouse tubular epithelial cells isolated from kidneys of healthy female C57 mice [as described previously in our lab (12)] were cultured in DMEM supplemented with l-glutamine (5 mM), penicillin (50 IU/ml), streptomycin (50 µg/ml), and 10% FCS.

#### Mouse Kidney Cortical Fibroblasts (mkCF)

Mouse kidney cortical fibroblasts were isolated from the kidneys of healthy male CD1 mice using a modified protocol described by Grimwood and Masterson (18). Briefly, cortical sections of kidneys from CD1 mice were minced and plated on gelatin-coated petridishes and incubated at 37°C for 72 h in DMEM high glucose supplemented with l-glutamine (5 mM), penicillin (50 IU/ml), streptomycin (50 µg/ml), 10 ml HEPES, and 20% FCS. The medium was replaced after 72 h and cultures allowed to grow for 10–14 days until a 75% confluent monolayer of fibroblast like cells was formed. Cells were further expanded and characterised for fibroblast lineage. Media was changed twice weekly to maintain cultures.

Fibroblasts were characterised through a series of inclusion/exclusion criteria according to their distinctive biochemical and morphological characteristics as reported previously (18). Cells were positive for established mesenchymal markers such as: vimentin and α-SMA, as well as fibroblast-specific protein 1 (FSP1), a marker of fibroblasts (19). In addition, expression of the endothelial/epithelial cell marker pan-cytokeratin was found to be negative, together with minimal expression of desmin (smooth muscle cell marker), therefore, confirming no contamination from these cells (18) (Figure S2 in Supplementary Material).

### Internalization of HLSC-EVs

For EV internalisation experiments, 2.5 × 10^4^ mkCF cells were seeded 24 h prior to treatment on gelatinised coverslips in a 24-well plate. Cells were then incubated for 6 h at 37°C with Dil labelled HLSC-EVs at a ratio of 4,000 EVs/cell in 2% FCS/DMEM high glucose medium. Post incubation, cells were fixed with 4% paraformaldehyde (Sigma, St. Louis, MO, USA) for 15 min at 4°C and permeabilised with 0.1% Triton-X100/PBS for 5 min at 4°C. Fluorescein isothiocyanate (FITC)-conjugated phalloidin dye (Thermo Fisher Scientific) (1:500) was then applied to the cells in 0.1% BSA/PBS for 1 h at room temperature. Post incubation, cells were washed twice with PBS for 5 min. Nuclei were stained with DAPI nuclear dye (Thermo Fisher Scientific) (1:5,000) for 10 min. Cover-slips were mounted on to glass slides and stored at 4°C until confocal microscopy.

### AA *In Vitro* Model

In order to study the effects of HLSC-EVs on mkCFs, an *in vitro* model of AA-induced fibrosis was set up. Briefly, 1.5 × 10^4^ mTECs pre-seeded in a 24-well cell culture inserts (1.0 µm pore) (Thermo Fisher Scientific) were exposed to 100 µM of AA for 4 h. Post incubation, cells were washed and cocultured with mkCF cells (2 × 10^4^ cells pre-seeded 24 h prior to the coculture) for 5 days at 37°C. For selected experiments, HLSC-EVs were added to fibroblasts cocultured with AA exposed mTECs at a concentration 50,000 EVs/cell. After 5 days of incubation, mkCFs were analysed for fibrotic gene expression by qRT-PCR. Fibroblasts cocultured with healthy mTECs served as controls.

### AAN *In Vivo* Model

**Figure 1:**
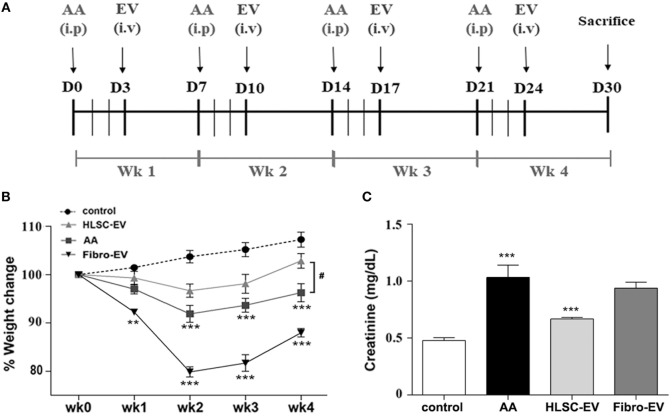
Human liver stem cell-derived extracellular vesicles (HLSC-EVs) exhibit antifibrotic effects in aristolochic acid nephropathy (AAN) *in vivo*. **(A)** Schematic overview of the experimental design for AAN *in vivo* showing the day of administration of aristolochic acid (AA), HLSC-EVs, or Fibro-EVs (EV) for 4 weeks (Wk). **(B)** Mice body weight was measured weekly prior to AA injections for 4 weeks and just before sacrifice. The body weight is represented as percentage weight change. A two-way analyses of variance (ANOVA) was performed with Bonferroni’s multi comparison test; data show mean ± SEM of nine mice per group. ***p < 0.001 AA vs control or Fibro-EVs vs control and **^#^***p* < 0.05 HLSC-EVs vs AA. **(C)** Plasma creatinine levels were assessed in mice treated with vehicle alone or mice injected with AA or AA mice injected with HLSC-EVs or Fibro-EVs (*n* = 9 mice per group). ***p < 0.001 AA vs control and **p < 0.01 HLSC-EVs vs AA. Data show mean ± SEM; a one-way ANOVA with Bonferroni’s multi comparison test was performed.

Animal studies were conducted in accordance with the National Institute of Health Guidelines for the Care and Use of Laboratory Animals. All procedures were approved by the Ethics Committee of the University of Turin and the Italian Health Ministry (authorisation number: 766/2016-PR). AAN was induced by injecting male NOD/SCID/IL2Rγ KO (NSG) mice (bred at the animal facility in the Molecular Biotechnology Centre) (6/8 weeks old; *n* = 12) with 4 mg/kg of AA (Santa Cruz Biotechnology, Santa Cruz CA, USA) on a weekly basis for 4 weeks intraperitoneally (Figure 1A). HLSC-EVs (*n* = 9) or fibroblast-derived EVs (Fibro-EVs; as negative control; *n* = 5) at a concentration of 1 × 10^10^ EVs/ml/mouse or vehicle alone (*n* = 5; as control) were injected 3 days after AA administration intravenously on a weekly basis (Figure 1A). After 4 weeks of treatments, mice were sacrificed and subjected to multi-parameter analyses as mentioned below. Five animals injected with PBS instead of AA served as controls. NSG immunodeficient mice were adopted for this study to prevent an immunogenic reaction during repeated administration of EVs.

### Plasma Creatinine and Body Weight

Kidney function of mice exposed to AA in the presence or absence of HLSC-EVs or Fibro-EVs was assessed by measuring blood plasma creatinine after sacrifice. Plasma creatinine was measured using a colourimetric microplate assay based on the Jaffe reaction (Quantichrom Creatinine Assay, BioAssay systems, Hayward, CA, USA) as per manufacturer’s protocol. Body weight of mice was also measured as a parameter for general health weekly before administering AA and at the end of the experiment prior to sacrifice.

### RNA Extraction and qRT-PCR

Total RNA was isolated from cells using TRIzol™ (Ambion, Thermofisher) followed by RNA extraction using the miRNeasy mini kit (Qiagen, Venlo, The Netherlands) according to the manufacturer’s protocol. Isolation of total RNA from mouse tissue was performed using the TRIzol™ as per manufacturer’s protocol. Briefly, mouse renal tissue was resuspended in 1 ml of TRIzol™ solution and homogenised in a Bullet blender (Next Advance Inc., New York, NY, USA) at a speed of 8 rpm for 3 min using 3.2 mm size zirconium pellets. Tubes were rotated on a rotator at 4°C for 30 min and centrifuged at 12,000 *g* for 15 min at 4°C. Supernatant from homogenised tissue was transferred to clean tubes and subjected to RNA isolation as mentioned above. Total RNA from HLSC-EVs was extracted using the All-in-one purification kit (Norgen, Biotek Corp.) as per manufacturer’s protocol. Isolated RNA was quantified using the NanoDrop2000 spectrophotometer (Thermo Fisher Scientific) and either used immediately or stored at −80°C until further use. For the synthesis of cDNA, 200 ng of RNA was retro-transcribed using the High Capacity cDNA reverse transcription kit (Applied Biosystems, Thermo Fisher Scientific) as per manufacturer’s protocol. qRT-PCR was performed using the StepOne Plus RT-PCR machine (Applied Biosystems) in 20 µl reaction mixtures containing: 4 ng of sample cDNA, specific oligonucleotide primers (Table 1) (MWG-Biotech, Eurofins Scientific, Brussels, Belgium) and Power SYBR Green PCR Master Mix (Applied Biosystems). *Gapdh* served as housekeeping gene. The data were analysed using ΔΔCt method.

**Table 1:** List of primers used for qRT-PCR.

Gene	Primer sequence
m_*Col1* Forward	ATC TCC TGG TGC TGA TGG AC
m_*Col1* Reverse	ACC TTG TTT GCC AGG TTC AC
m_*Tgfb1* Forward	CGA AAG CCC TGT ATT CCG TCT
m_*Tgfb1* Reverse	GCA ACA ATT CCTGGC GTT ACC
m_α*-Sma* Forward	CTG ACA GAG GCA CCA CTG AA
m_α*-Sma* Reverse	CAT CTC CAG AGT CCA GCA CA
m_*Ltbp1* Forward	GGA GCC CGA AGT GGT AAC AG
m_*Ltbp1* Reverse	GAA TAG TTG AAA CCC CTG GGG
m_*Gapdh* Forward	TGT CAA GCT CAT TTC CTG GTA TGA
m_*Gapdh* Reverse	TCT TAC TCC TTG GAG GCC ATG T

### Renal Histopathology

Renal histology was assessed by staining 5 µm-thick kidney sections with haematoxylin and eosin (H&E, Merck, Darmstadt, Germany) or Masson’s Trichrome stain and analysed through microscopic examination. Tubular necrosis was assessed in non-overlapping fields (10/section) with an objective of 40× (high power field, HPF). Interstitial fibrosis was quantified by measuring collagenous fibrotic areas stained in blue (sections stained with Masson’s trichrome) in 10 random cortical fields/section from images taken at a magnification of 20× using multiphase image analysis with ImageJ software version 1.49s (20).

Immunohistochemistry was performed as described previously (9). Briefly, 5 µm-thick kidney sections were subjected to antigen retrieval, blocking and labelling with monoclonal anti-proliferating cell nuclear antigen (PCNA) (Santa Cruz Biotechnology) at a concentration of 1:400. Secondary horseradish peroxide conjugated antibody (Pierce, Rockford, IL, USA) was applied and the slides analysed *via* microscopy. Histological scoring was performed by counting the number of positive nuclei per HPF (40×) in 10 random sections of the renal cortex.

Immunofluorescence was performed on 5 µm-thick cryostat sections. Briefly, sections were stained with mouse anti-CD45 (Biorbyt, San Francisco CA, USA), mouse anti-S100A4 [fibroblast specific protein 1 (FSP-1)] (Abcam, Cambridge, MA, USA), or mouse anti-collagen 1 (Abcam) for 2 h at 4°C. This was followed by FITC or Texas red conjugated secondary antibody staining for 1 h at room temperature. Sections were mounted and analysed *via* confocal microscopy. Sections labelled with secondary antibodies only, served as controls. All the above image analyses were performed in a double blind format.

### Fibrosis Array and miRnome Array

In order to elucidate the regulation of pro-fibrotic genes in mice treated with AA in the presence or absence of HLSC-EVs, total RNA extracted from kidneys of experimental mice were analysed using the Fibrosis RT^2^ Profiler PCR array (PAMM-120Z, Qiagen) as per manufacturer’s protocol. Analyses were performed using the online software provided by the manufacturer with global normalisation. A total of three mice per experimental group were subjected to array analyses. Furthermore, total RNA from the same mice was subjected to miRNA analyses using the QuantiMir™ mouse miRNome microRNA profiler array (RA670A-1, Systems Biosciences, Palo Alto, CA, USA) according to manufacturer’s protocol. In addition, total RNA from mkCF from the *in vitro* experiments were subjected to miRNA analyses using the QuantiMir™ mouse miRNome microRNA profiler array. Only selected microRNAs (miRNAs) upregulated or downregulated by HLSC EVs following AA treatment in mice (*in vivo*) were assessed.

### Bioinformatic Analyses

Data obtained from the mouse miRnome array were subjected to bioinformatic analyses. Briefly, gene target prediction analyses and biological pathway analyses was performed using MirWalk v2.0 (21) and Panther gene ontology analyses tool (22) available online. Only biological processes of selected genes showing a *p* value < 0.05 were considered as significantly enriched.

### Electron Microscopy

Transmission electron microscopy was performed by loading HLSC-EVs onto 200 mesh nickel formvar carbon coated grids (Electron Microscopy Science) for 20 min. This was followed by fixation in a 2.5% glutaraldehyde/2% sucrose solution. Following repeated washings in distilled water, samples were negatively stained with NanoVan (Nanoprobes, Yaphank) and examined by a Jeol JEM 1010 electron microscope (Joel, USA).

### Statistical Analyses

Data analyses were performed using GraphPad Prism 6.0. Results are expressed as mean ± SD or SEM where indicated. Statistical analyses were performed by employing: Student’s *t-*test, one way ANOVA, or two-way ANOVA with a multi comparison test where appropriate. A *p* value of <0.05 was considered statistically significant.

## Results

### HLSC-EVs Prevent Development of CKD

**Figure 2:**
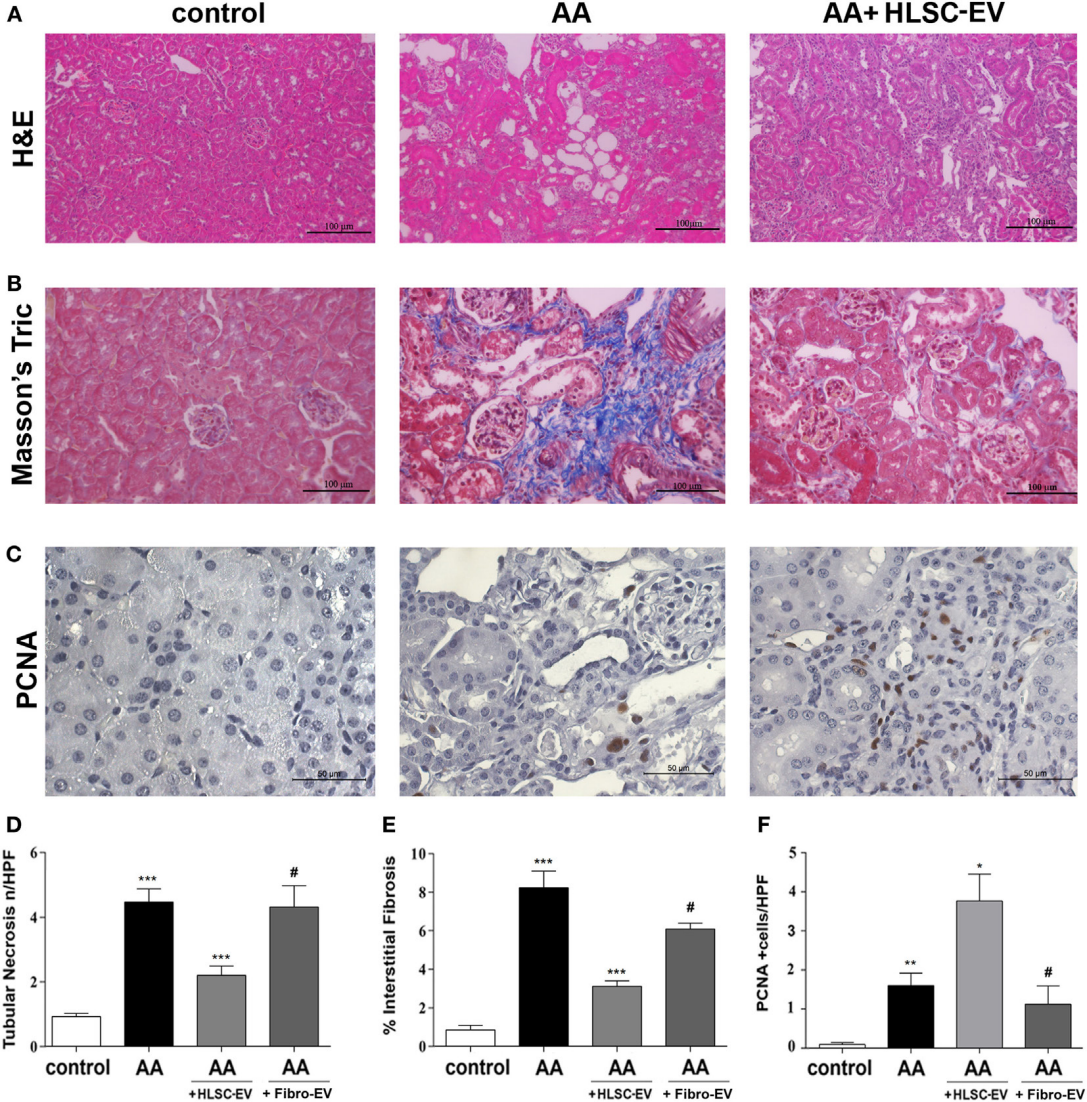
Histological analyses of aristolochic acid nephropathy (AAN) *in vivo* model. **(A)** Representative micrographs of H&E-stained renal tissue from healthy mice injected with vehicle alone (control), or mice injected with aristolochic acid (AA), or AA mice treated with human liver stem cell-derived extracellular vesicles (HLSC-EVs). **(B)** Representative micrographs of Masson’s trichrome stained renal sections from control, AA, or HLSC-EVs treated AA mice. The blue stain represents collagen fibres considered to be a marker for interstitial fibrosis. Original magnification at 400×. **(C)** Representative micrographs of proliferating cell nuclear antigen (PCNA) stained renal tissue of control, AA, or AA mice treated with HLSC-EVs original magnification at 400×. **(D)** Histological score of tubular necrosis in AAN mice experimental groups. Mice treated with AA had significantly elevated levels of tubular necrosis, which was alleviated on treatment with HLSC-EVs. No significant reduction in tubular necrosis was observed in mice treated with Fibro-EVs. Data represent mean ± SD of tubular necrosis observed under high power field (original magnification: 400×). A one-way analyses of variance (ANOVA) with Bonferroni’s multi comparison test was performed. ***p < 0.001 AA vs control or HLSC-EVs vs AA, ^#^*p* < 0.01 Fibro-EVs vs HLSC-EVs. **(E)** Histological quantification of interstitial fibrosis in AAN mice experimental groups by multiphase image analysis of 10 fields per section. Data represent mean ± SD; a one-way ANOVA with Bonferroni’s multi comparison test was performed. ***p < 0.001 AA vs control, **p < 0.01 HLSC-EVs vs AA, ^#^*p* < 0.01 Fibro-EVs vs HLSC-EVs. **(F)** Histological score of PCNA positive cells in AAN mice experimental groups observed under high power with an original magnification of 400×. An increase in PCNA positive cells (marker for proliferation) was observed in HLSC-EVs treated mice renal tissue. No significant increase in PCNA was observed in AA mice treated with Fibro-EVs compared to control. Data represent mean ± SD. A one-way ANOVA with Bonferroni’s multi comparison test was performed. **p < 0.01 AA vs control, or Fibro-EVs vs HLSC-EVs, *p < 0.05 HLSC-EVs vs AA, ^#^*p* < 0.01 Fibro-EVs vs HLSC-EVs (*n* = 9 mice per group).

The body weight of mice measured weekly prior to AA injection (see *in vivo* model schematic, Figure 1A) was significantly reduced with respect to controls injected with the vehicle alone. The difference in weight was statistically significant after the second injection (week 2) and continued to remain low until sacrificing at week 4 (Figure 1B). On the other hand, body weight of AA mice injected with HLSC-EVs increased gradually reaching a significant difference at week 4 compared to mice treated with AA alone. In contrast, mice treated with Fibro-EVs lost more weight compared to mice injected with AA only (Figure 1B). Serum creatinine levels were significantly increased in AA treated mice at week 4 with respect to controls. In contrast, mice treated with HLSC-EVs had significantly reduced levels of serum creatinine compared to mice treated with AA alone or with Fibro-EV, which served as a negative control (Figure 1C). Assessment of morphological alterations revealed severe tubular damage in mice injected with AA. The major changes included diffuse degeneration of the proximal tubular epithelium together with hyaline cast formation and tubular necrosis (Figures 2A,D). However, mice treated with HLSC-EVs had significantly lower number of hyaline casts and necrotic tubules compared to mice injected with AA alone (Figures 2A,D). The histological score obtained by quantifying tubular necrosis in kidney sections of every experimental group showed a statistically significant increase in AA mice (compared to control mice) and a significant reduction (protection) in mice treated with HLSC-EVs (compared to AA mice) (Figure 2D). No significant reduction in tubular necrosis was observed in mice treated with Fibro-EVs (Figure 2D).

Kidney fibrosis, as demonstrated by Masson’s trichrome staining, showed an increase in tubular damage and interstitial fibrosis 4 weeks after AA injection (Figures 2B,E), which was significantly reduced by treatment with HLSC-EVs (Figures 2B,E). Some reduction of fibrosis was also observed in Fibro-EV treated mice. However, the reduction observed was significantly less effective compared to HLSC-EV treatment. PCNA (a marker of cell proliferation) analysis showed a significant increase in PCNA-positive cells in AA animals treated with HLSC-EVs but not with Fibro-EVs (Figures 2C,F).

**Figure 3:**
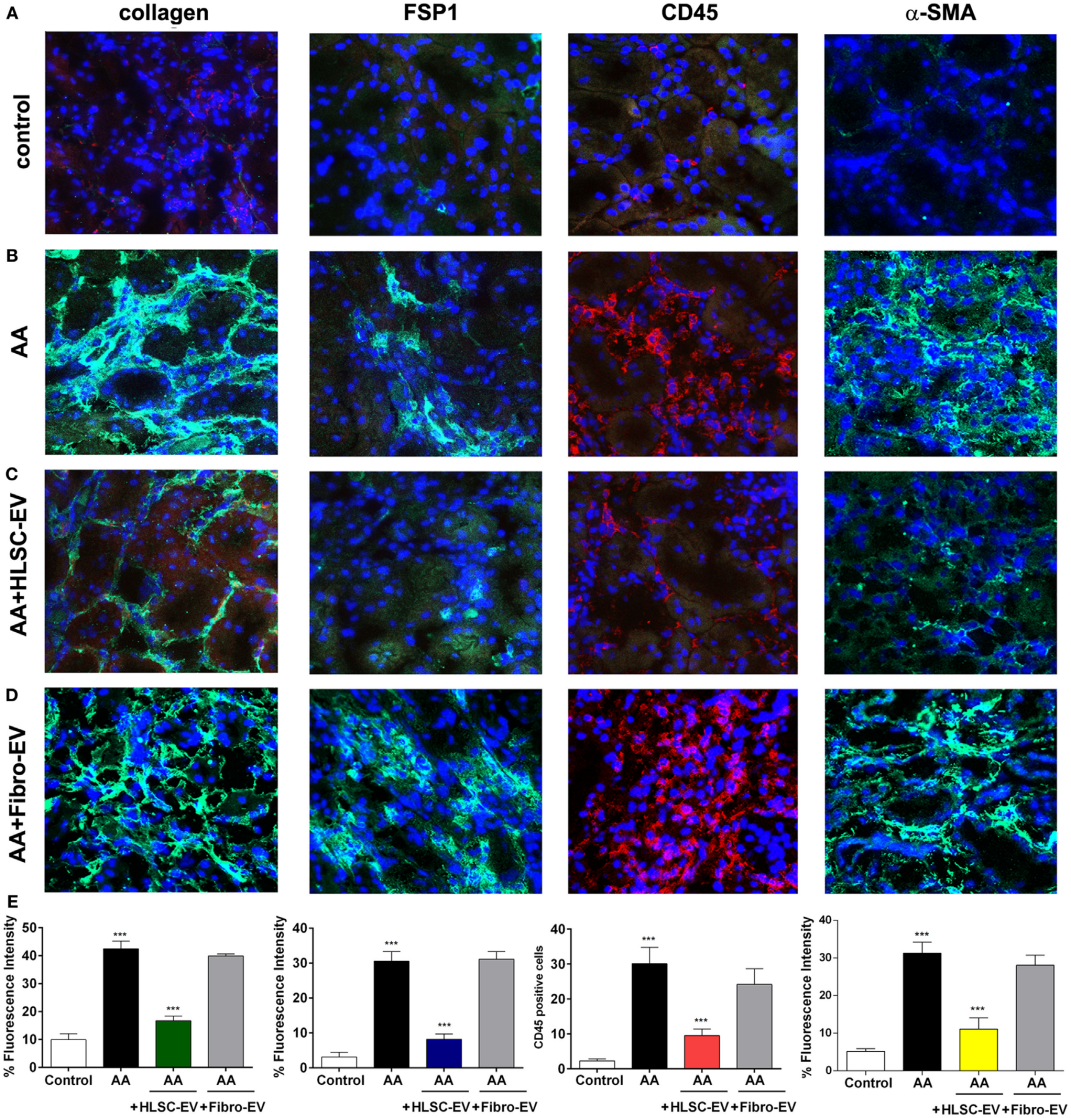
Immunofluorescent staining of kidneys from mice treated with aristolochic acid (AA). Kidney cryo-sections from healthy mice **(A)**, mice treated with AA **(B)**, AA mice treated with HLSC-EVs **(C)**, or Fibro-EVs **(D)** were stained for collagen 1a1, fibroblast-specific protein 1 (FSP-1), CD45, and α-SMA to identify presence of tissue fibrosis, fibroblasts, and inflammatory cells (Original magnification: 200×). **(E)** Histograms depicts the fluorescence intensity of collagen, FSP-1, α-SMA, and quantification of cells positive for CD45 in mouse kidney cryo-sections from AAN mice experimental groups. Data represent mean ± SD of the fluorescence intensity or cells positive per high power field measured from 10 images taken at random from six samples per treatment (*n* = 6 mice). ***p < 0.001 AA vs control or HLSC-EVs vs AA. No significant differences were observed between AA vs Fibro-EVs.

Immunohistochemical staining of kidney cryo-sections showed that mice treated with AA had significantly elevated numbers of CD45, FSP-1, and α-SMA positive cells as well as type 1 collagen deposition compared to controls (Figures 3A,B). However, on treating mice with HLSC-EVs, expression levels of CD45, FSP-1, α-SMA, and Collagen 1 were markedly reduced (Figure 3C). Morphometric analyses revealed a significant difference between the three experimental groups of animals, showing a protective effect of HLSC-EVs on AA induced injury (Figure 3E). However, no significant reduction in the expression levels of CD45, FSP-1, α-SMA, and Collagen 1 were observed in mice treated with Fibro-EVs (Figures 3D,E).

### HLSC-EVs Downregulate Pro-Fibrotic Genes in Kidneys of AA-Treated Mice

**Figure 4:**
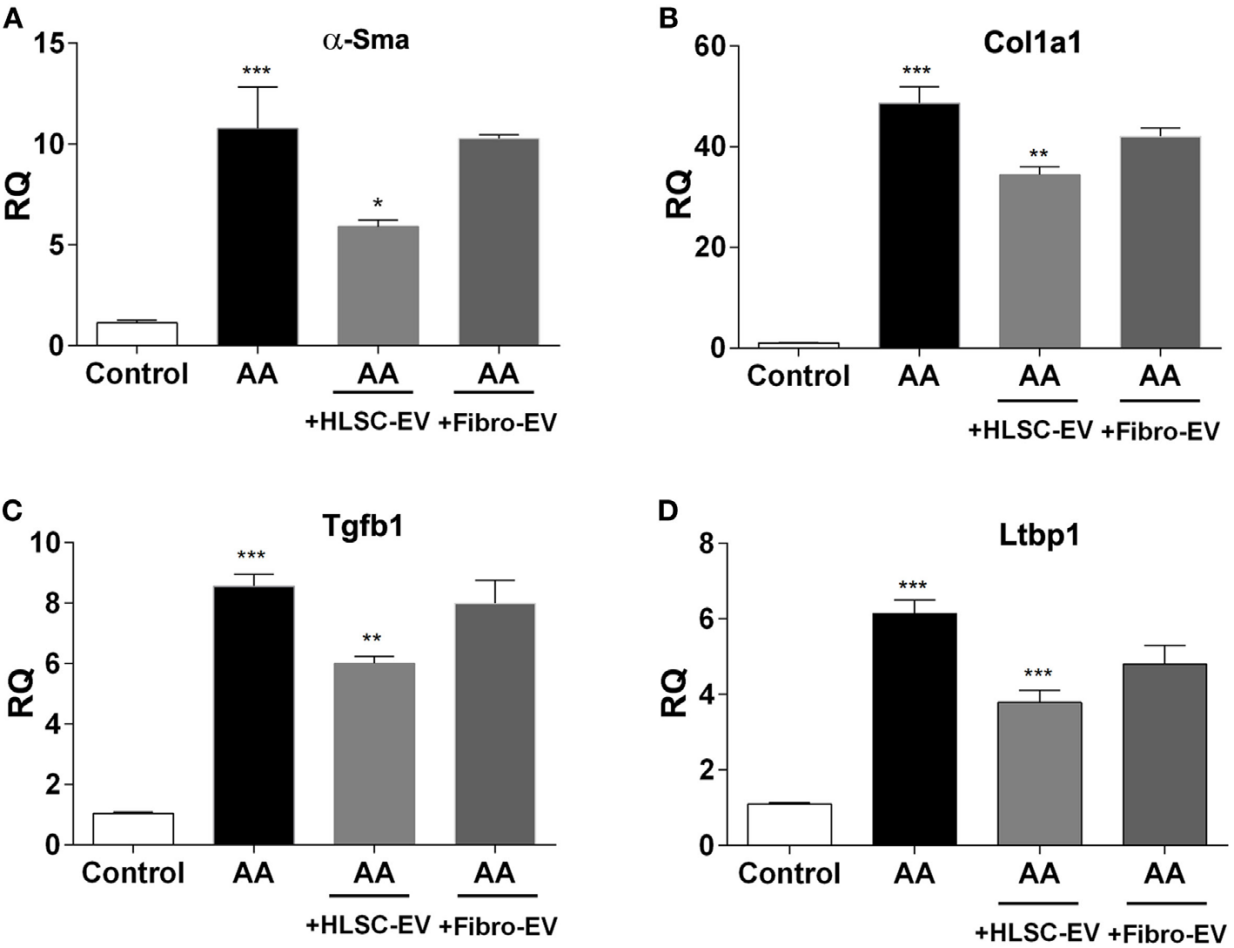
Human liver stem cell-derived extracellular vesicles (HLSC-EVs) downregulate pro-fibrotic genes in mice treated with aristolochic acid (AA). Gene expression levels of α*-Sma* **(A)**, *Col1a1* **(B)**, *Tgfb1* **(C)**, and *Ltbp1* **(D)** in mice treated with vehicle alone (control), mice treated with AA, AA mice treated with HLSC-EVs or Fibro-EVs. Data show mean ± SD of *n* = 7 mice per treatment. ***p < 0.001 AA vs control, *p < 0.05, **p < 0.01, ***p < 0.001 HLSC-EVs vs AA. No significant differences were observed between AA vs Fibro-EVs.

Kidney tissue obtained from mice treated with AA in the presence or absence of EVs was subjected to RNA isolation. Real-time PCR showed mice treated with AA had significantly upregulated levels of the pro-fibrotic genes: alpha smooth muscle actin (α*-Sma*) (Figure 4A), *Col1a1* (collagen 1a1) (Figure 4B), and *Tgfb1* (TGFβ 1) (Figure 4C). Furthermore, we also observed an upregulation of the gene latent-transforming growth factor beta-binding protein 1 (*Ltbp1*) that codes for the protein (LTBP1), which is responsible for activating TGFβ1 from its latent form (Figure 4D). In contrast, mice treated with HLSC-EVs had a significant reduction in the expression levels of all three pro-fibrotic genes (Figures 4A–C). In addition, a downregulation of *Ltbp1* gene was also observed in mice treated with HLSC-EVs (Figure 4D). Mice treated with Fibro-EVs showed no significant downregulation of pro-fibrotic genes as well as *Ltbp1* (Figures 4A–D).

### HLSC-EVs Downregulate Pro-Fibrotic Genes in Fibroblasts *In Vitro*

**Figure 5:**
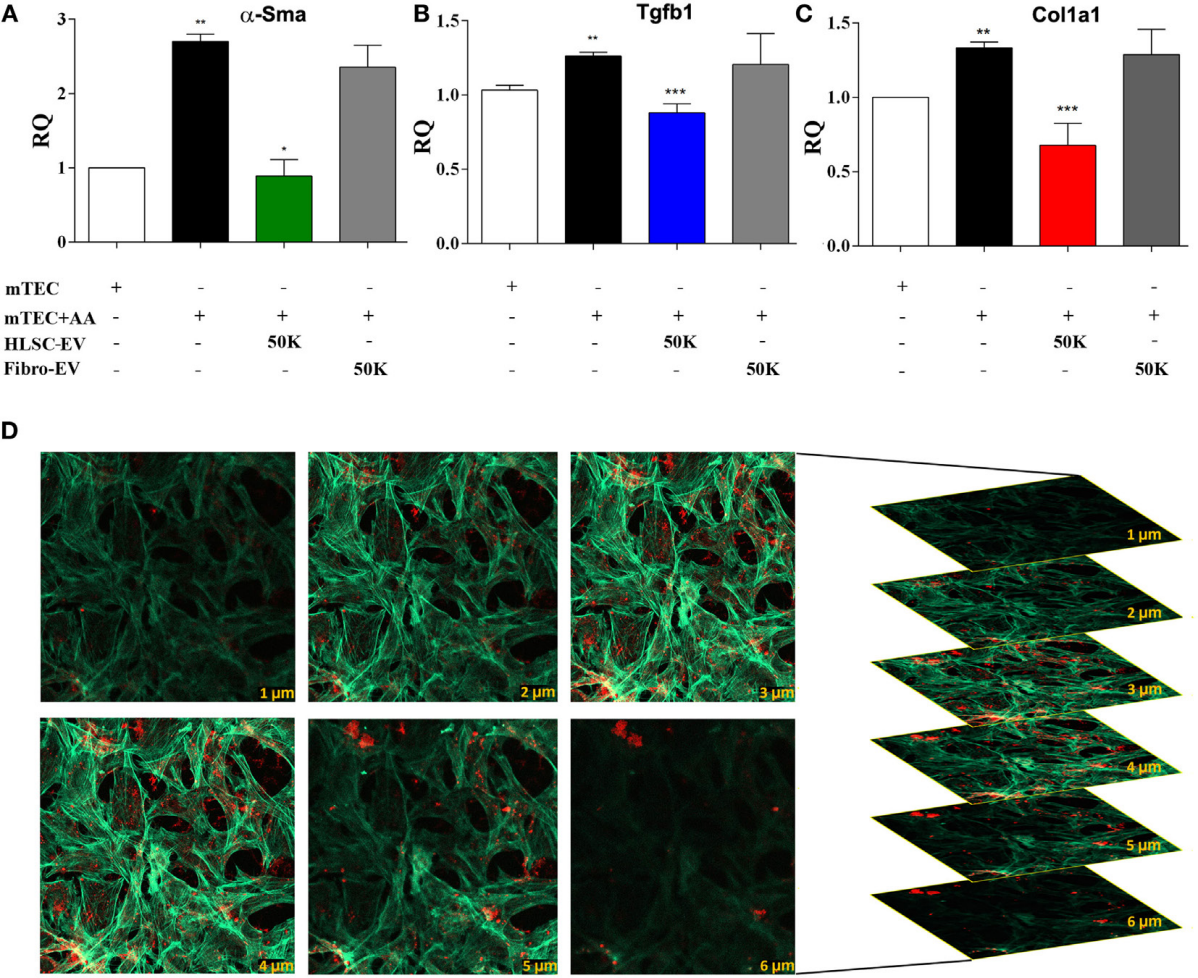
Human liver stem cell-derived extracellular vesicles (HLSC-EVs) downregulate pro-fibrotic genes in mkCF in an *in vitro* model of aristolochic acid nephropathy. mTECs pre-treated with 100 µM aristolochic acid (AA) for 4 h were cocultured with mouse fibroblasts in the presence or absence of HLSC-EVs or Fibro-EVs (50,000 EVs/cell) for 5 days at 37°C. Post experimental analyses revealed an upregulation of the fibrotic markers: **(A)** α*-sma*, **(B)** *Tgfb1*, and **(C)** *Col1a1* in mkCF cocultured with AA-treated mTECs (AA). Treatment with HLSC-EVs, but not Fibro-EVs, significantly downregulated all three genes compared to control. The data represent mean ± SEM of three independent experiments performed in quadruplicate. *p < 0.05, **p < 0.01, ***p < 0.001 mTEC + AA vs Control, or HLSC-EVs vs mTEC + AA. No significant differences were observed between mTEC + AA vs Fibro-EVs. A one-way analyses of variance with Bonferroni’s multi comparison test was performed. **(D)** Representative confocal microscopy depicting the uptake of 1 × 10^10^ Dil dye-labelled HLSC-EVs by mkCF after 6 h of co-incubation at 37°C. *Z* stack analyses shows the presence of EVs within the cytoplasm (phalloidin staining, green) of the cells indicating an effective internalisation of vesicles (in red); scale bar = 50 μm. Data represent one of the three experiments performed with similar results.

In order to study the effects of HLSC-EVs on renal fibroblasts, an *in vitro* model was set up whereby mTECs pre-exposed to AA were cocultured with fibroblasts in a transwell system. A significant rise in α*-Sma, Tgfb1, Col1a1* expression levels was observed in fibroblasts exposed to mTEC pre-treated with AA compared to control (Figures 5A–C). This upregulation of pro-fibrotic genes was significantly reduced in the presence of HLSC-EVs, but not Fibro-EVs (Figures 5A–C). Furthermore, internalisation of HLSC-EVs by mkCF cells (co-incubation for 6 h) was confirmed by *z*-stack imagery obtained through confocal microscopy (Figure 5D).

### Mouse miRNome Array

In order to analyse the regulation of miRNAs in kidneys of mice treated with AA in the presence or absence of HLSC-EVs, the mouse miRNome miRNA profiling kit was adopted. Analyses of the data revealed that out of 709 miRNAs analysed, 38 miRNAs were upregulated and 92 were downregulated in mice treated with AA (Table S1 in Supplementary Material). Furthermore, an upregulation of 37 miRNAs and downregulation of 47 miRNAs was also observed in AA mice treated with HLSC-EVs (Table S2 in Supplementary Material).

**Figure 6:**
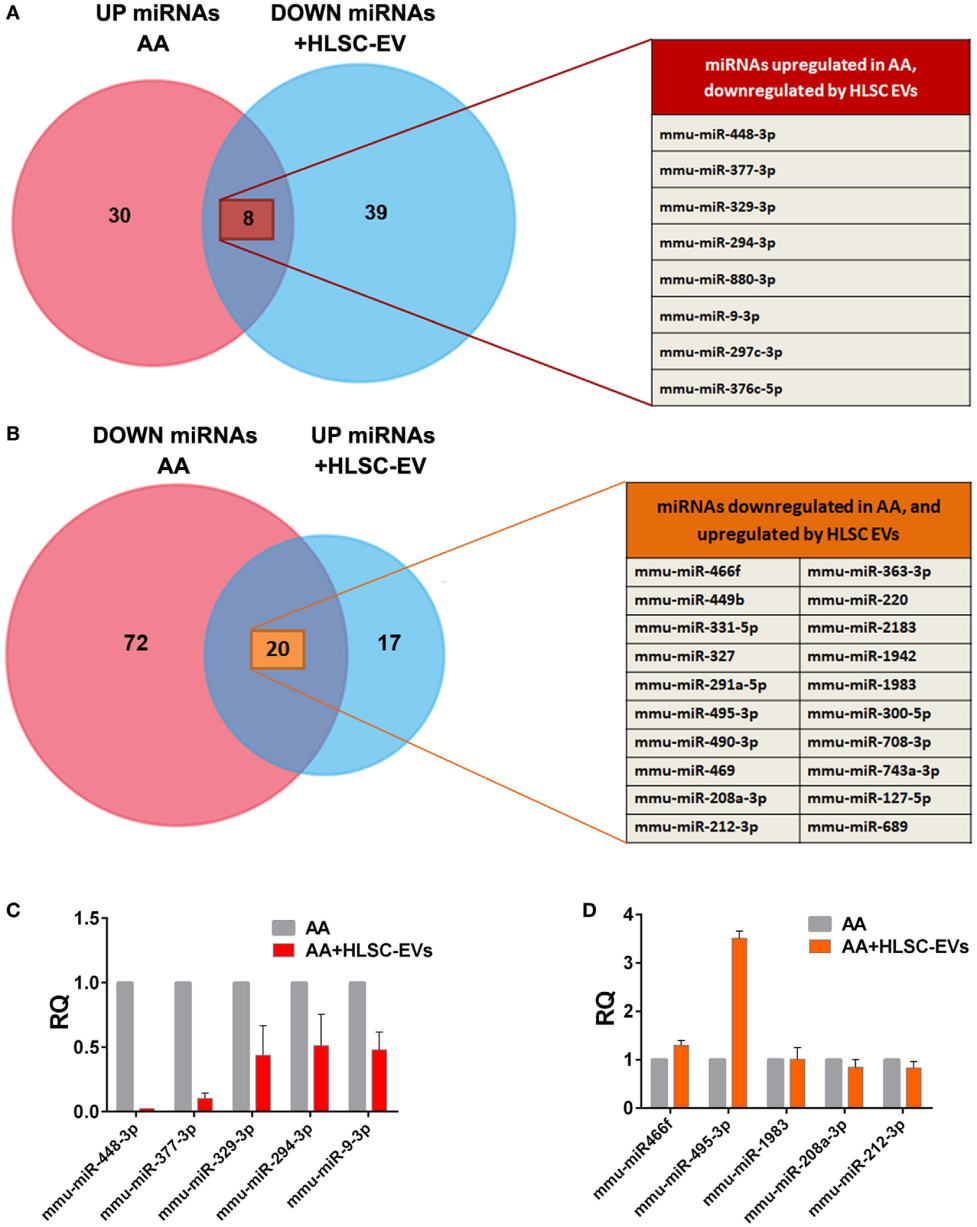
Venn diagram comparing microRNAs (miRNAs) regulated by HLSC-EVs treatment *in vivo* and *in vitro*. **(A)** 38 miRNAs were upregulated in mice treated with aristolochic acid (AA) and 47 miRNAs were downregulated in AA mice treated with human liver stem cell-derived extracellular vesicles (HLSC-EVs). Eight miRNAs were common between the two groups. **(B)** 92 miRNAs were downregulated in mice treated with AA and 37 were upregulated on treating the AA mice with HLSC-EVs. Twenty miRNAs were common between the two groups. *n* = 3 mice/treatment. Expression levels of miRNAs regulated by HLSC-EVs *in vivo* were verified in mkCF cocultured with AA pre-treated mTECs. Five were found to be downregulated *in vitro* **(C)**, and two miRNAs upregulated **(D)**. The data represent mean ± SEM of three independent experiments performed in quadruplicate.

Comparing the list of miRNAs upregulated in AA mice with the list of miRNAs downregulated on treating the mice with HLSC-EVs, 8 miRNAs were found to be common (Figure 6A). Furthermore, a comparison was also made to identify miRNAs that were downregulated by AA and upregulated when treating them with HLSC-EVs. The comparison revealed 20 miRNAs to be common between the two groups (Figure 6B). Expression levels of the 28 miRNAs regulated by HLSC-EVs were verified in fibroblasts from the *in vitro* system. It was observed that out of the 28 miRNAs regulated by HLSC-EVs *in vivo*, 5 were downregulated and 2 upregulated in fibroblasts cocultured with AA pre-treated mTECs *in vitro* (Figures 6C,D).

**Figure 7:**
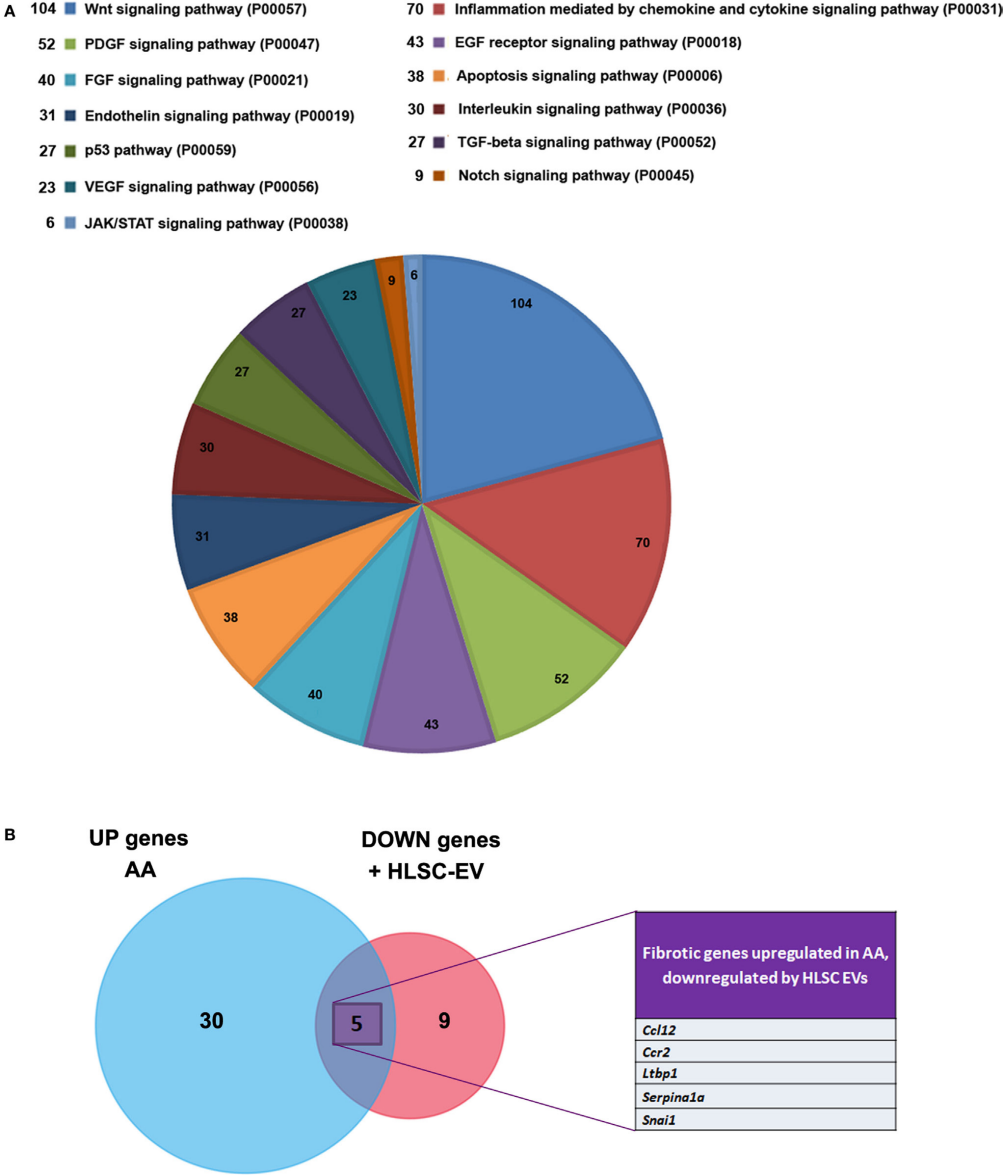
Panther pathway analyses of microRNAs (miRNAs) regulated by human liver stem cell-derived extracellular vesicles (HLSC-EVs) and Venn diagram illustrating the pro-fibrotic genes. **(A)** Pie chart showing various pathways involved in the process of aristolochic acid (AA) kidney injury as described in Table 2. Pathway analyses of miRNAs regulated by HLSC-EVs in AA mice showed the regulation of wingless-related integration site signalling pathway, platelet derived growth factor signalling pathway, fibroblast growth factor, and TGFβ signalling pathway. **(B)** Venn diagram of pro-fibrotic genes regulated in mice treated with AA and in AA mice treated with HLSC-EVs showed 35 genes were upregulated in mice treated with AA and 14 genes were downregulated in AA mice treated with HLSC-EVs out of which 5 genes were upregulated by AA.

After identifying common miRNAs that inversely correlated between mice treated with AA and AA mice treated with HLSC-EVs, we sought to investigate the genes and pathways that were regulated by these miRNAs. Mirwalk analyses identified over 7,000 predicted genes that were regulated by these miRNAs (data not shown). On analysing these genes using Panther gene ontology software online, 141 pathways were linked with the miRNA targeted genes inputted. These pathways were ranked according to the number of genes involved per pathway and the top 36 pathways were selected based on the cut off of >5 genes/pathway (Table 2). Interestingly, out of the pathways identified, quite a few have been implicated in fibrosis including, the wingless-related integration site (WNT) signalling pathway, inflammatory cytokine and chemokine pathway, platelet derived growth factor, fibroblast growth factor (FGF), and TGFβ signalling pathways (Figure 7A; Table 2). Out of interest, the WNT signalling pathway had the highest number of the predicted miRNA target genes.

**Table 2:** Panther pathway enrichment analyses.

Pathways predicted by PANTHER online meta-analyses	No of genes involved
**Wnt signalling pathway (P00057)**	**104**
**Inflammation mediated by chemokine and cytokine signalling pathway (P00031)**	**70**
Angiogenesis (P00005)	58
Cadherin signalling pathway (P00012)	57
Integrin signalling pathway (P00034)	**53**
**Platelet-derived growth factor signalling pathway (P00047)**	**52**
**Epidermal growth factor receptor signalling pathway (P00018)**	**43**
**Fibroblast growth factor signalling pathway (P00021)**	**40**
**Apoptosis signalling pathway (P00006)**	**38**
Ras Pathway (P04393)	34
**Endothelin signalling pathway (P00019)**	**31**
T cell activation (P00053)	31
**Interleukin signalling pathway (P00036)**	**30**
Cytoskeletal regulation by Rho GTPase (P00016)	27
**p53 pathway (P00059)**	**27**
**TGF-beta signalling pathway (P00052)**	**27**
**VEGF signalling pathway (P00056)**	**23**
B cell activation (P00010)	21
Oxidative stress response (P00046)	19
p38 MAPK pathway (P05918)	18
Beta1 adrenergic receptor signalling pathway (P04377)	17
Beta2 adrenergic receptor signalling pathway (P04378)	17
PI3 kinase pathway (P00048)	17
Toll receptor signalling pathway (P00054)	17
Dopamine receptor mediated signalling pathway (P05912)	15
Insulin/IGF pathway-protein kinase B signalling cascade (P00033)	13
Hypoxia response *via* HIF activation (P00030)	12
FAS signalling pathway (P00020)	**11**
Histamine H1 receptor mediated signalling pathway (P04385)	11
Interferon-gamma signalling pathway (P00035)	11
Transcription regulation by bZIP transcription factor (P00055)	11
Angiotensin II-stimulated signalling through G proteins and beta-arrestin (P05911)	10
Insulin/IGF pathway-mitogen activated protein kinase kinase/MAP kinase cascade (P00032)	10
Histamine H2 receptor mediated signalling pathway (P04386)	9
**Notch signalling pathway (P00045)**	**9**
**JAK/STAT signalling pathway (P00038)**	**6**

*Pathways regulated by microRNAs downregulated by human liver stem cell-derived extracellular vesicles in aristolochic acid mice. Out of 141 pathways identified, 36 pathways have been selected on the basis of number of genes involved*.

*Bold red indicated the pathways and gene numbers implicated in kidney fibrosis*.

In order to understand the regulation of genes involved in fibrosis in the current AAN model, mice treated with AA in the presence or absence of HLSC-EVs were analysed through a Fibrosis RT^2^ Profiler PCR array (Qiagen). Out of 84 genes in the array, 35 were found to be upregulated in mice treated with AA and 14 genes downregulated in HLSC-EV treated mice (Figure 7B; Table 3). On comparing the genes between the two experimental groups, five genes were found to be common (upregulated in AA treated mice and downregulated in HLSC-EV treated mice) (Figure 7B; Table 3). Altogether, these results suggest that HLSC-EVs could mediate the progression and development of fibrotic events through the regulation of miRNAs and pro-fibrotic genes.

**Table 3:** Regulation of pro-fibrotic genes in AA mice and AA mice treated with HLSC-EVs.

Genes upregulated in AA mice		Genes downregulated in HLSC-EV mice
*Acta2*	*Mmp14*	***Ccl12***
*Cav1*	*Mmp2*	***Ccr2***
*Ccl11*	*Mmp3*	*Egf*
***Ccl12***	*Mmp8*	*Fasl*
*Ccl3*	*Mmp9*	*Ifng*
***Ccr2***	*Myc*	*Il13*
*Col1a2*	*Plat*	*Il13ra2*
*Col3a1*	***Serpina1a***	*Inhbe*
*Dcn*	*Serpine1*	***Ltbp1***
*Edn1*	***Snai1***	*Plg*
*Grem1*	*Tgfb1*	***Serpina1a***
*Hgf*	*Tgfb2*	*Smad6*
*Il1b*	*Thbs1*	***Snai1***
*Itga2*	*Thbs2*	*Stat1*
*Itga3*	*Timp1*	
*Jun*	*Timp2*	
*Lox*	*Tnf*	
***Ltbp1***		

*Thirty five genes were upregulated in mice treated with aristolochic acid (AA). Fourteen genes were downregulated in AA mice treated with human liver stem cell-derived extracellular vesicles (HLSC-EVs). Five genes were common between the two lists (highlighted in red)*.

## Discussion

In the current study, we demonstrate that HLSC-EVs prevent the development of interstitial fibrosis and tubular necrosis, favours renal regeneration, and reduces the infiltration of inflammatory immune cells in a CKD mouse model of AAN. Furthermore, there is also an improvement in kidney function as observed through significantly low levels of plasma creatinine.

The beneficial effects of bone marrow MSC-derived EVs on AKI was first reported by Bruno et al. (12). They showed that a single intravenous dose of MSC-EVs at the peak of the damage was sufficient to alleviate morphological and functional impairment in a glycerol induced model of AKI (12). Thereafter, several studies have reported the beneficial effects of stem cell-derived EVs in various models of AKI. For instance, in a lethal model of cisplatin induced AKI, the authors observed that a single intravenous dose of MSC-EVs improved renal function and morphology, therefore, improving overall survival rate of injured mice (23). In addition, this effect was further enhanced by the administration of multiple doses of MSC-EVs (23). In another study, human umbilical cord blood derived MSC-EVs injected through the caudal vein reduced damage in a cisplatin-induced AKI model *in vivo* and *in vitro* (24). A similar regenerative and therapeutic effect was also observed by Burger et al. (25) in their model of AKI, whereby EVs from endothelial precursor cells were injected through the jugular vein (25). There is, therefore, sufficient evidence to show that MSC derived EVs exhibit therapeutic effects in various models of AKI regardless the source or route of administration. Although, plenty of research has been published over the years on stem cell derived EVs in AKI, very few studies can be found reporting the effect of EVs in CKD (23, 26). Nonetheless, these studies have shown that MSC derived EVs may prevent CKD progression (13, 26–29). For instance, in a rat model of ischemia–reperfusion-induced (IRI) chronic kidney injury, Gatti et al. showed that MSC-EVs, not only improved kidney function by reducing elevated levels of blood urea nitrogen and creatinine, but also prevented further progression to fibrosis (13). Furthermore, a subsequent study in a similar model of IRI reported a reduction in the infiltration of inflammatory cells mainly through the suppression of fractalkine (CX3CL1) mediated by MSC-EVs (30). Apart from single doses of EVs, multiple doses have also proven to be effective in CKD. For example, several injections of urine derived stem cell EVs not only prevented apoptosis of podocytes and tubular epithelial cells, but also favoured proliferation of glomerular endothelial cells, therefore, exhibiting an overall regenerative effect in a model of Type 1 diabetes (31). Although, in the past, we have reported how HLSC-EVs promote regeneration and recovery in AKI (9), no studies have been performed investigating the biological activity of HLSC-EVs in CKD. Here, we report for the first time the effect of HLSC-EVs in a severe AAN model of CKD in NSG mice.

One of the key features of CKD is loss of kidney function reflected by the rise in blood creatinine levels as observed in various models of CKD including AAN (2, 13, 28, 32). The findings of the current study are in line with these reports as mice treated with AA had significantly elevated levels of creatinine. We also observed that treatment with HLSC-EVs was able to reduce blood creatinine in injured mice significantly, therefore, confirming the ability of HLSC-EVs to improve renal function as observed previously in our model of AKI (9). Another key factor in the progression of CKD is interstitial inflammation characterised by infiltrating immune and inflammatory cells regardless the cause of injury (5). Depierreux for instance reported the presence of lymphocytic infiltration in biopsies of patients with AAN (33). Later on, Pozdzik et al. (34, 35) confirmed this in human patients with AAN as well as in a rat AAN model whereby an influx of activated mononuclear cells and cytotoxic T lymphocytes was observed in the renal interstitium (34, 35). A similar finding was also obtained in another study, whereby the authors reported infiltration of CD45 positive immune cells in their mouse model of AAN (36). In the present study, an infiltration of CD45-positive cells was also observed 30 days after initiating the damage with AA. We, therefore, speculate that, these cells are likely to be monocytes and neutrophils as NSG mice lack lymphocytes and natural killer cells and have macrophages and dendritic cells, which are defective due to their genetic background (37). Interestingly, AA mice treated with HLSC-EVs had lower counts of CD45 mononuclear cells, therefore, suggesting an immune/anti-inflammatory role of these HLSC-EVs not been reported previously in CKD.

Another key feature of CKD is tubular damage and hyaline cast formation, which has also been characterised in various models of AAN (36, 38, 39). The development of proximal tubular necrosis and cast formation was recapitulated in this model representing the predominant lesions observed. Interestingly, treatment with HLSC-EVs significantly reduced tubular necrosis in our model compared to treatment with vehicle alone. In addition, a marked increase in proliferating cells confirmed by PCNA staining in HLSC-EV-treated mice kidneys demonstrate regeneration and recovery of damaged renal tissue. Indeed, these tissue healing properties of stem cell-derived EVs and HLSC-EVs, in particular, have been reported in an AKI model previously (9). However, the observation of similar effects extended to CKD is novel.

Irrespective to the cause of injury, interstitial fibrosis is the final common process that defines CKD and is characterised by proliferation of resident fibroblasts and progressive accumulation of ECM primarily collagen (5, 36). In this study, we observed an increase in the infiltration/proliferation of fibroblasts in the renal interstitium, which correlated with an increase in Collagen type 1 level in mice treated with AA. These findings were consistent with what has been reported in other models of AAN (34–36, 40), in particular, by Huang et al. (36) who also reported an increase of FSP-1 positive cells in a mouse model of chronic AAN. Moreover, AA mice treated with HLSC-EVs had a significantly reduced percentage of fibroblasts, as well as collagen deposition in the renal interstitium. A similar result was also observed at a molecular level whereby, expression levels of the pro-fibrotic genes α*-Sma, Col1a1*, and *Tgfb1* were downregulated in mice renal tissue treated with HLSC-EVs. This result was supported by *in vitro* data whereby HLSC-EVs downregulated pro-fibrotic genes mentioned above, in renal cortical fibroblasts cocultured with AA exposed mTECs. This finding, therefore, suggests a novel role of HLSC-EVs as mediators of fibroblast activation.

TGFβ is considered to be a major driver of ECM synthesis, inhibition of ECM degradation and activation of myofibroblasts, all of which lead to the development of fibrosis (5). Furthermore, the upregulation of this cytokine has been reported in various models of AAN (34, 40–42). We, therefore, investigated the molecular events involved around the TGFβ signalling pathway that lead to the development of fibrosis, and whether, the healing properties of HLSC-EVs observed in the current model could be due to the regulation of the genes governed by this pathway. Thirty genes related to fibrosis were identified to be upregulated in mice treated with AA. Some of these genes such as: *Cav1, Dcn, Grem1, Ltbp1, Tgfb1*, etc., belong to the TGFβ superfamily including two genes (*Jun* and *Myc*) that code for transcription factors involved in the pathway. On the other hand, 14 pro-fibrotic genes in total were downregulated in mice treated with HLSC-EVs, five of which were initially upregulated in AA mice (Table 2). These 14 genes regulate various pathways that are dysregulated during fibrosis including: cytokine and chemokine pathways, growth factors, regulation of the ECM, etc.

Out of the five pro-fibrotic genes downregulated by HLSC-EV treatment, *Ccr2* and *Ccl12* code for inflammatory chemokines, which have a receptor–ligand relationship (43). Furthermore, they have also been implicated in fibrosis. For instance, Moore et al. (43) in their study showed that mice genetically deficient in *Ccr2* gene were protected from lung fibrosis. Furthermore, neutralisation of *Ccl12* in wild-type mice significantly protected them from FITC-induced lung fibrosis by preventing the recruitment of fibrocytes (43). Similarly, in another study, the blocking of *Ccr2*, either through gene silencing or inhibitors reduced renal interstitial fibrosis induced by unilateral ureter obstruction in mice (44). Furthermore, this blockade of *Ccr2* resulted in the downregulation of TGFβ, and type 1 collagen both at a molecular and protein level (44) as was observed in our model. Another gene that was downregulated through the treatment of HLSC-EVs was snail family zinc finger 1 (*Snai1*). This gene codes for the transcription factor SNAI1 that is very well known to mediate biological processes involved in renal fibrogenesis including EMT of tubular epithelial cells, cell cycle arrest, and inflammation (45). Furthermore, it has also been reported to be upregulated in the kidney tissue of patients with various types of progressive nephropathy (46).

Latent-transforming growth factor beta-binding protein 1 (*LTBP1*) is a gene that codes for the LTBP1 protein. TGFβ is normally secreted as a latent form comprised of three components: the mature TGFβ dimer, the latency-associated peptide, and LTBP (47). LTBP1 not only facilitates the formation and release of latent TGFβ but is also involved in the activation of the cytokine. Furthermore, both TGFβ and LTBP1 were found to be upregulated in patients with idiopathic lung fibrosis (48). Consistent with this report, we also observed an upregulation of *Ltbp1* expression levels together with *Tgfb1* in mice renal tissue exposed to AA. However, interestingly, mice treated with HLSC-EVs showed lower expression levels of both genes. As mentioned previously, as LTBP1 is important for the activation of TGFβ, this finding could provide one explanation of the mechanism of action of HLSC-EV mediated downregulation of TGFβ1.

MicroRNAs are small, non-coding RNAs that regulate gene expression by suppressing or degrading target mRNA at a posttranscriptional level (49). They have been implicated in various physiological and pathological processes including fibrosis (49). We, therefore, investigated the regulation of miRNAs in mice injured with AA and AA mice treated with HLSC-EVs. After identifying the common miRNAs that were inversely correlated between AA mice and mice treated with HLSC-EV, we analysed the genes and pathways that were regulated by these miRNAs. Inputting the data into bioinformatic analyses databases online, we identified over 7,000 genes that were regulated by these miRNAs (data not shown). Furthermore, Panther pathway analyses linked these genes to 141 pathways out of which the top 36 pathways (ranked according to the number of genes involved per pathway; Table 2) have been reported here. Out of the pathways identified, quite a few have been implicated in fibrosis such as the WNT signalling pathway (50), inflammatory cytokine, and chemokine pathways (51), as well as PDGF, FGF, and TGFβ pathways (52).

Taken together, we demonstrate for the first time the healing properties of HLSC-EVs in a model of CKD. Our data indicate that multiple injections of HLSC-EVs exhibit a regenerative, anti-fibrotic, and anti-inflammatory role in a mouse model of AAN characterised by severe tubular epithelial cell necrosis and interstitial fibrosis. Furthermore, downregulation of pro-fibrotic genes and modulation of miRNAs that regulate the fibrotic pathways could be a mechanism through which HLSC-EVs exert their biological activity. These findings may promote the development of new therapeutic strategies involving HLSC-EVs in CKD.

## Ethics Statement

Animal studies were conducted in accordance with the National Institute of Health Guidelines for the Care and Use of Laboratory Animals. All procedures were approved by the Ethics Committee of the University of Turin and the Italian Health Ministry (authorisation number: 766/2016-PR).

## Author Contributions

SK, MBHS, and GC contributed conception and design of the study, acquisition, analyses and interpretation of data, as well as drafting the manuscript. MC, EP, MT, and MCD contributed towards acquisition and analyses of data. MFB and CT contributed towards interpretation of data, manuscript preparation, and final approval. All authors contributed towards the manuscript revision, as well as reading and approving the submitted version.

## Conflict of Interest Statement

CT (Unicyte AG) is employed by commercial company and contributed to the study as a researcher. GC is member of the Scientific Advisory Board of Unicyte AG. MBHS, MCD, CT, and GC are named inventors in related patents. The other authors declare that they have no competing interests.

## Abbreviations

CKD, chronic kidney disease; AA, aristolochic acid; AAN, aristolochic acid nephropathy; HLSC, human liver stem cell; TGFb, transforming growth factor beta; α-Sma, alpha smooth muscle actin; LTBP1, latent TGFb binding protein 1; EMT, epithelial to mesenchymal transition; ECM, extracellular matrix; AKI, acute kidney injury; DMSO, dimethyl sulfoxide; α-MEM, alpha minimum essential medium; EBM, endothelial basal medium; mTEC, mouse tubular epithelial cell; mkCF, mouse kidney cortical fibroblast; WNT, wingless-related integration site; PDGF, platelet-derived growth factor; EGF, epidermal growth factor; FGF, fibroblast growth factor.

## References

[1] Coresh J Selvin E Stevens LA Manzi J Kusek JW Eggers P Prevalence of chronic kidney disease in the United States. JAMA (2007) 298(17):2038–47. 10.1001/jama.298.17.2038 17986697

[2] Sun D Bu L Liu C Yin Z Zhou X Li X Therapeutic effects of human amniotic fluid-derived stem cells on renal interstitial fibrosis in a murine model of unilateral ureteral obstruction. PLoS One (2013) 8(5):e65042 10.1371/journal.pone.0065042 23724119 PMC3665750

[3] Nicholson ML McCulloch TA Harper SJ Wheatley TJ Edwards CM Feehally J Early measurement of interstitial fibrosis predicts long-term renal function and graft survival in renal transplantation. Br J Surg (1996) 83(8):1082–5. 10.1002/bjs.1800830813 8869307

[4] Leaf IA Duffield JS. What can target kidney fibrosis? Nephrol Dial Transplant (2017) 32(Suppl_1):i89–97. 10.1093/ndt/gfw388 28391346

[5] Jadot I Decleves AE Nortier J Caron N. An integrated view of aristolochic acid nephropathy: update of the literature. Int J Mol Sci (2017) 18(2):E297. 10.3390/ijms18020297 28146082 PMC5343833

[6] Matsui F Babitz SA Rhee A Hile KL Zhang H Meldrum KK. Mesenchymal stem cells protect against obstruction-induced renal fibrosis by decreasing STAT3 activation and STAT3-dependent MMP-9 production. Am J Physiol Renal Physiol (2017) 312(1):F25–32. 10.1152/ajprenal.00311.2016 27760767 PMC5283885

[7] Herrera MB Bruno S Buttiglieri S Tetta C Gatti S Deregibus MC Isolation and characterization of a stem cell population from adult human liver. Stem Cells (2006) 24(12):2840–50. 10.1634/stemcells.2006-0114 16945998

[8] Herrera MB Fonsato V Bruno S Grange C Gilbo N Romagnoli R Human liver stem cells improve liver injury in a model of fulminant liver failure. Hepatology (2013) 57(1):311–9. 10.1002/hep.25986 22829291

[9] Herrera Sanchez MB Bruno S Grange C Tapparo M Cantaluppi V Tetta C Human liver stem cells and derived extracellular vesicles improve recovery in a murine model of acute kidney injury. Stem Cell Res Ther (2014) 5(6):124. 10.1186/scrt514 25384729 PMC4446072

[10] de Jong OG van Balkom BW Schiffelers RM Bouten CV Verhaar MC. Extracellular vesicles: potential roles in regenerative medicine. Front Immunol (2014) 5:608. 10.3389/fimmu.2014.00608 25520717 PMC4253973

[11] Giebel B On the function and heterogeneity of extracellular vesicles. Ann Transl Med (2017) 5(6):150 10.21037/atm.2017.02.14 28462230 PMC5395490

[12] Bruno S Grange C Deregibus MC Calogero RA Saviozzi S Collino F Mesenchymal stem cell-derived microvesicles protect against acute tubular injury. J Am Soc Nephrol (2009) 20(5):1053–67. 10.1681/ASN.2008070798 19389847 PMC2676194

[13] Gatti S Bruno S Deregibus MC Sordi A Cantaluppi V Tetta C Microvesicles derived from human adult mesenchymal stem cells protect against ischaemia-reperfusion-induced acute and chronic kidney injury. Nephrol Dial Transplant (2011) 26(5):1474–83. 10.1093/ndt/gfr015 21324974

[14] Collino F Deregibus MC Bruno S Sterpone L Aghemo G Viltono L Microvesicles derived from adult human bone marrow and tissue specific mesenchymal stem cells shuttle selected pattern of miRNAs. PLoS One (2010) 5(7):e11803. 10.1371/journal.pone.0011803 20668554 PMC2910725

[15] Herrera Sanchez MB Previdi S Bruno S Fonsato V Deregibus MC Kholia S Extracellular vesicles from human liver stem cells restore argininosuccinate synthase deficiency. Stem Cell Res Ther (2017) 8(1):176. 10.1186/s13287-017-0628-9 28750687 PMC5531104

[16] Cavallari C Ranghino A Tapparo M Cedrino M Figliolini F Grange C Serum-derived extracellular vesicles (EVs) impact on vascular remodeling and prevent muscle damage in acute hind limb ischemia. Sci Rep (2017) 7(1):8180. 10.1038/s41598-017-08250-0 28811546 PMC5557987

[17] Kowal J Arras G Colombo M Jouve M Morath JP Primdal-Bengtson B Proteomic comparison defines novel markers to characterize heterogeneous populations of extracellular vesicle subtypes. Proc Natl Acad Sci U S A (2016) 113(8):E968–77. 10.1073/pnas.1521230113 26858453 PMC4776515

[18] Grimwood L Masterson R. Propagation and culture of renal fibroblasts. Methods Mol Biol (2009) 466:25–37. 10.1007/978-1-59745-352-3_3 19148603

[19] Strutz F Okada H Lo CW Danoff T Carone RL Tomaszewski JE Identification and characterization of a fibroblast marker: FSP1. J Cell Biol (1995) 130(2):393–405. 10.1083/jcb.130.2.393 7615639 PMC2199940

[20] Schneider CA Rasband WS Eliceiri KW NIH image to ImageJ: 25 years of image analysis. Nat Methods (2012) 9(7):671–5. 10.1038/nmeth.2089 22930834 PMC5554542

[21] Dweep H Gretz N Sticht C. miRWalk database for miRNA-target interactions. Methods Mol Biol (2014) 1182:289–305. 10.1007/978-1-4939-1062-5_25 25055920

[22] Thomas PD Campbell MJ Kejariwal A Mi H Karlak B Daverman R PANTHER: a library of protein families and subfamilies indexed by function. Genome Res (2003) 13(9):2129–41. 10.1101/gr.772403 12952881 PMC403709

[23] Bruno S Grange C Collino F Deregibus MC Cantaluppi V Biancone L Microvesicles derived from mesenchymal stem cells enhance survival in a lethal model of acute kidney injury. PLoS One (2012) 7(3):e33115. 10.1371/journal.pone.0033115 22431999 PMC3303802

[24] Zhou Y Xu H Xu W Wang B Wu H Tao Y Exosomes released by human umbilical cord mesenchymal stem cells protect against cisplatin-induced renal oxidative stress and apoptosis in vivo and in vitro. Stem Cell Res Ther (2013) 4(2):34 10.1186/scrt194 23618405 PMC3707035

[25] Burger D Vinas JL Akbari S Dehak H Knoll W Gutsol A Human endothelial colony-forming cells protect against acute kidney injury: role of exosomes. Am J Pathol (2015) 185(8):2309–23. 10.1016/j.ajpath.2015.04.010 26073035

[26] Grange C Iampietro C Bussolati B. Stem cell extracellular vesicles and kidney injury. Stem Cell Investig (2017) 4:90. 10.21037/sci.2017.11.02 29270416 PMC5723738

[27] He J Wang Y Sun S Yu M Wang C Pei X Bone marrow stem cells-derived microvesicles protect against renal injury in the mouse remnant kidney model. Nephrology (Carlton) (2012) 17(5):493–500. 10.1111/j.1440-1797.2012.01589.x 22369283

[28] Nagaishi K Mizue Y Chikenji T Otani M Nakano M Konari N Mesenchymal stem cell therapy ameliorates diabetic nephropathy via the paracrine effect of renal trophic factors including exosomes. Sci Rep (2016) 6:34842. 10.1038/srep34842 27721418 PMC5056395

[29] Cantaluppi V Gatti S Medica D Figliolini F Bruno S Deregibus MC Microvesicles derived from endothelial progenitor cells protect the kidney from ischemia-reperfusion injury by microRNA-dependent reprogramming of resident renal cells. Kidney Int (2012) 82(4):412–27. 10.1038/ki.2012.105 22495296

[30] Zou X Zhang G Cheng Z Yin D Du T Ju G Microvesicles derived from human Wharton’s Jelly mesenchymal stromal cells ameliorate renal ischemia-reperfusion injury in rats by suppressing CX3CL1. Stem Cell Res Ther (2014) 5(2):40. 10.1186/scrt428 24646750 PMC4055103

[31] Jiang ZZ Liu YM Niu X Yin JY Hu B Guo SC Exosomes secreted by human urine-derived stem cells could prevent kidney complications from type I diabetes in rats. Stem Cell Res Ther (2016) 7:24. 10.1186/s13287-016-0287-2 26852014 PMC4744390

[32] Decleves AE Jadot I Colombaro V Martin B Voisin V Nortier J Protective effect of nitric oxide in aristolochic acid-induced toxic acute kidney injury: an old friend with new assets. Exp Physiol (2016) 101(1):193–206. 10.1113/EP085333 26442795

[33] Depierreux M Van DB Vanden Houte K Vanherweghem JL. Pathologic aspects of a newly described nephropathy related to the prolonged use of Chinese herbs. Am J Kidney Dis (1994) 24(2):172–80. 10.1016/S0272-6386(12)80178-8 8048421

[34] Pozdzik AA Salmon IJ Husson CP Decaestecker C Rogier E Bourgeade MF Patterns of interstitial inflammation during the evolution of renal injury in experimental aristolochic acid nephropathy. Nephrol Dial Transplant (2008) 23(8):2480–91. 10.1093/ndt/gfn140 18385385

[35] Pozdzik AA Berton A Schmeiser HH Missoum W Decaestecker C Salmon IJ Aristolochic acid nephropathy revisited: a place for innate and adaptive immunity? Histopathology (2010) 56(4):449–63. 10.1111/j.1365-2559.2010.03509.x 20459552

[36] Huang L Scarpellini A Funck M Verderio EA Johnson TS. Development of a chronic kidney disease model in C57BL/6 mice with relevance to human pathology. Nephron Extra (2013) 3(1):12–29. 10.1159/000346180 23610565 PMC3617971

[37] Shultz LD Lyons BL Burzenski LM Gott B Chen X Chaleff S Human lymphoid and myeloid cell development in NOD/LtSz-scid IL2R gamma null mice engrafted with mobilized human hemopoietic stem cells. J Immunol (2005) 174(10):6477–89. 10.4049/jimmunol.174.10.6477 15879151

[38] Debelle FD Vanherweghem JL Nortier JL. Aristolochic acid nephropathy: a worldwide problem. Kidney Int (2008) 74(2):158–69. 10.1038/ki.2008.129 18418355

[39] Debelle FD Nortier JL De Prez EG Garbar CH Vienne AR Salmon IJ Aristolochic acids induce chronic renal failure with interstitial fibrosis in salt-depleted rats. J Am Soc Nephrol (2002) 13(2):431–6. 11805172 10.1681/ASN.V132431

[40] Pozdzik AA Salmon IJ Debelle FD Decaestecker C Van den Branden C Verbeelen D Aristolochic acid induces proximal tubule apoptosis and epithelial to mesenchymal transformation. Kidney Int (2008) 73(5):595–607. 10.1038/sj.ki.5002714 18094681

[41] Zhou L Fu P Huang XR Liu F Chung AC Lai KN Mechanism of chronic aristolochic acid nephropathy: role of Smad3. Am J Physiol Renal Physiol (2010) 298(4):F1006–17. 10.1152/ajprenal.00675.2009 20089673

[42] Wang Y Zhang Z Shen H Lu Y Li H Ren X TGF-beta1/Smad7 signaling stimulates renal tubulointerstitial fibrosis induced by AAI. J Recept Signal Transduct Res (2008) 28(4):413–28. 10.1080/10799890802176741 18702012

[43] Moore BB Murray L Das A Wilke CA Herrygers AB Toews GB. The role of CCL12 in the recruitment of fibrocytes and lung fibrosis. Am J Respir Cell Mol Biol (2006) 35(2):175–81. 10.1165/rcmb.2005-0239OC 16543609 PMC2643255

[44] Kitagawa K Wada T Furuichi K Hashimoto H Ishiwata Y Asano M Blockade of CCR2 ameliorates progressive fibrosis in kidney. Am J Pathol (2004) 165(1):237–46. 10.1016/S0002-9440(10)63292-0 15215179 PMC1618531

[45] Simon-Tillaux N Hertig A. Snail and kidney fibrosis. Nephrol Dial Transplant (2017) 32(2):224–33. 10.1093/ndt/gfw333 28186539

[46] Ohnuki K Umezono T Abe M Kobayashi T Kato M Miyauchi M Expression of transcription factor Snai1 and tubulointerstitial fibrosis in progressive nephropathy. J Nephrol (2012) 25(2):233–9. 10.5301/JN.2011.8449 21725924

[47] Schlotzer-Schrehardt U Zenkel M Kuchle M Sakai LY Naumann GO. Role of transforming growth factor-beta1 and its latent form binding protein in pseudoexfoliation syndrome. Exp Eye Res (2001) 73(6):765–80. 10.1006/exer.2001.1084 11846508

[48] Lepparanta O Sens C Salmenkivi K Kinnula VL Keski-Oja J Myllarniemi M Regulation of TGF-beta storage and activation in the human idiopathic pulmonary fibrosis lung. Cell Tissue Res (2012) 348(3):491–503. 10.1007/s00441-012-1385-9 22434388

[49] O’Reilly S. MicroRNAs in fibrosis: opportunities and challenges. Arthritis Res Ther (2016) 18:11. 10.1186/s13075-016-0929-x 26762516 PMC4718015

[50] Tan RJ Zhou D Zhou L Liu Y Wnt/b-catenin signaling and kidney fibrosis. Kidney Int Suppl (2011) (2014) 4(1):84–90. 10.1038/kisup.2014.16 26312156 PMC4536962

[51] Lee SB Kalluri R. Mechanistic connection between inflammation and fibrosis. Kidney Int Suppl (2010) 78(119):S22–6. 10.1038/ki.2010.418 21116313 PMC4067095

[52] Liu M Ning X Li R Yang Z Yang X Sun S Signalling pathways involved in hypoxia-induced renal fibrosis. J Cell Mol Med (2017) 21(7):1248–59. 10.1111/jcmm.13060 28097825 PMC5487923

