# Supplementary material for: Human Liver Stem Cell-Derived Extracellular Vesicles Prevent Aristolochic Acid-Induced Kidney Fibrosis
Source: Front Immunol. 2018 Jul 19;9:1639. doi: 10.3389/fimmu.2018.01639 (PMC6060249; doi:10.3389/fimmu.2018.01639)
Supplement: Supplementary file 1 [file Data_Sheet_1.docx]

Supplementary Material

**Human Liver Stem cell derived Extracellular Vesicles prevents aristolochic acid induced kidney fibrosis**

Sharad Kholia^1,3#^, Maria Beatriz Herrera Sanchez^2,3#^, Massimo Cedrino^2,3^, Elli Papadimitrou^3,5^, Marta Tapparo^1,3^, Maria Chiara Deregibus^2,3^, Maria Felice Brizzi^1^, Ciro Tetta^4^ and Giovanni Camussi^1*^

*** Correspondence:** Prof. Giovanni Camussi:[giovanni.camussi@unito.it](mailto:giovanni.camussi@unito.it)

^#^ Equally contributed

# Supplementary Figures and Tables

## Supplementary Figures

**

**

**Supplementary Figure 1:** **Characterization of HLSC-EVs.** **(A)**. Nanoparticle tracking analyses showing the size distribution and quantity of HLSC-EVs purified by Iodixanol/sucrose floating (15% gradient). **(B)** Representative transmission electron microscopy showing HLSC-EVs (original magnification x150,000; bar=100nm). **(C)** Western blot analysis confirming the expression of CD63, CD81 and TSG101 in HLSC-EVs purified by; a. differential ultracentrifugation; b. Iodixanol/sucrose floating (15% gradient).

**
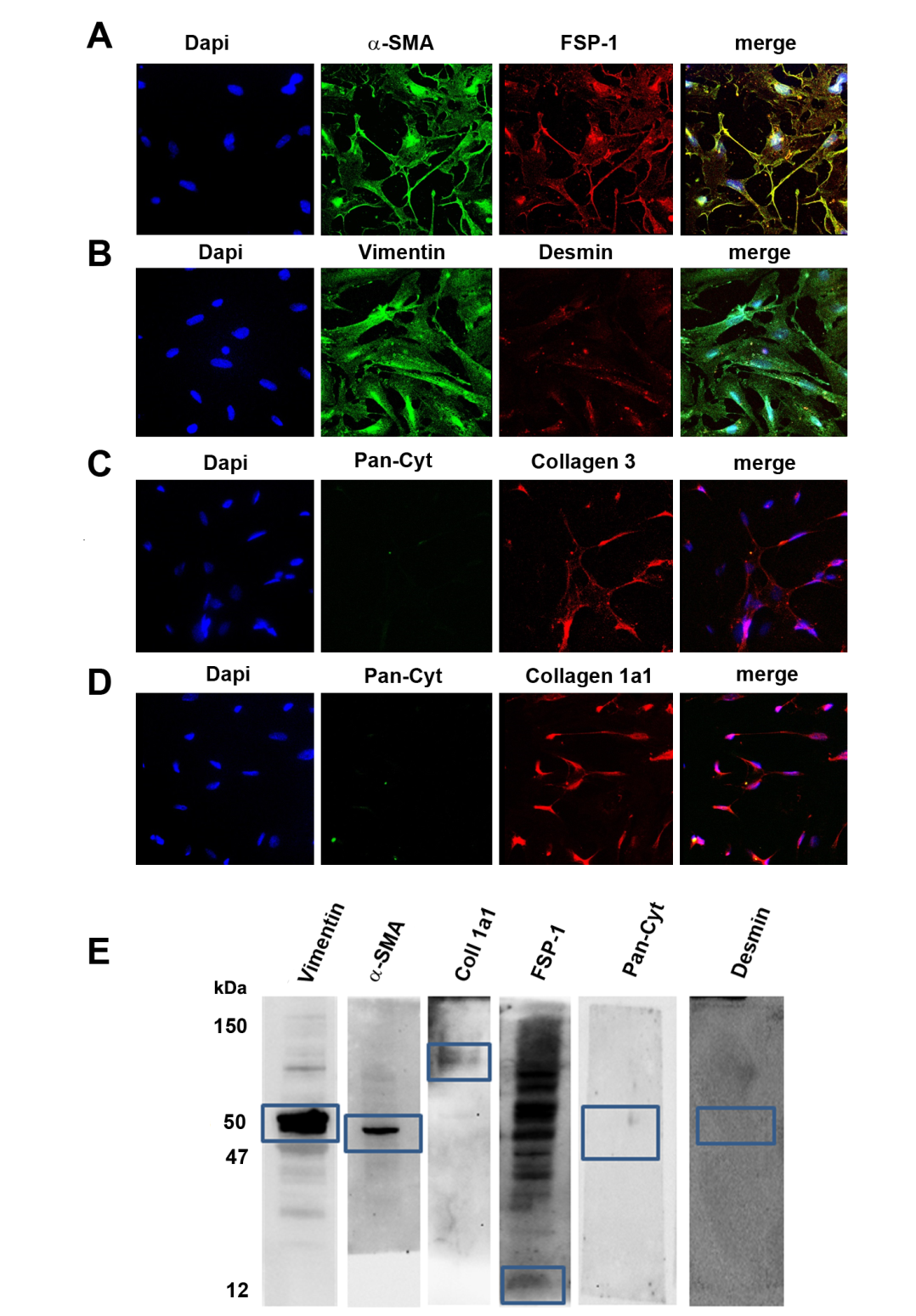
**

**Supplementary Figure 2: Characterisation of mouse renal cortical fibroblasts (mkCF).** Immunofluorescence microscopy confirming the expression of fibroblast markers: α-SMA and FSP-1 **(A)**, Vimentin **(B)**, Collagen 3 **(C)**, and Collagen 1a1 **(D)**. Cells were negative for: the endothelial/epithelial marker Pan-Cytokeratin (Pan-Cyt) **(C, D)**. Low expression of the smooth muscle cell marker Desmin was observed **(B)**. **(E)** Western blot analysis confirmed the cells to be positive for the markers: Vimentin, α-SMA, Collagen 1a1 and FSP-1, and negative for the endothelial/epithelial marker Pan-Cyt and Desmin.

| **miRNA downregulated in AA mice** | | | |
| --- | --- | --- | --- |
| mmu-miR-380-3p | mmu-miR-217-5p | mmu-miR-293-5p | mmu-miR-451a |
| mmu-miR-540-3p | mmu-miR-220 | mmu-miR-300-5p | mmu-miR-212-3p |
| mmu-miR-509-3p | mmu-miR-290a-5p | mmu-miR-374b-3p | mmu-miR-411-5p |
| mmu-miR-466f | mmu-miR-302b-3p | mmu-miR-376a-5p | mmu-miR-383-5p |
| mmu-miR-465c-5p | mmu-let-7f-5p | mmu-miR-463-5p | mmu-miR-669d-5p |
| mmu-miR-465b-5p | mmu-miR-1904 | mmu-miR-467e-3p | mmu-miR-365-3p |
| mmu-miR-465a-3p | mmu-miR-878-3p | mmu-miR-470-3p | mmu-miR-363-3p |
| mmu-miR-449b | mmu-miR-883a-5p | mmu-miR-708-3p | mmu-miR-346-5p |
| mmu-miR-345-3p | mmu-miR-1192 | mmu-miR-881-5p | mmu-miR-680 |
| mmu-miR-331-5p | mmu-miR-1894-3p | mmu-miR-2133 | mmu-miR-686 |
| mmu-miR-327 | mmu-miR-1901 | mmu-miR-2134 | mmu-miR-719 |
| mmu-miR-323-3p | mmu-miR-1903 | mmu-miR-2135 | mmu-miR-718 |
| mmu-miR-669g | mmu-miR-2183 | mmu-miR-2143 | mmu-miR-707 |
| mmu-miR-291a-5p | mmu-miR-1931 | mmu-miR-186-3p | mmu-miR-688 |
| mmu-miR-302d-3p | mmu-miR-1933-5p | mmu-miR-154-3p | mmu-miR-693-5p |
| mmu-miR-665-3p | mmu-miR-1942 | mmu-miR-1964-3p | mmu-miR-874-3p |
| mmu-miR-18b-5p | mmu-miR-1945 | mmu-miR-99b-3p | mmu-miR-15a-3p |
| mmu-miR-125b-1-3p | mmu-miR-1951 | mmu-miR-873a-5p | mmu-miR-702-3p |
| mmu-miR-495-3p | mmu-miR-1952 | mmu-miR-802-5p | mmu-miR-669k-3p |
| mmu-miR-494-3p | mmu-miR-1953 | mmu-miR-881-3p | mmu-miR-201-5p |
| mmu-miR-493-3p | mmu-miR-1960 | mmu-miR-758-3p | mmu-miR-208a-3p |
| mmu-miR-490-3p | mmu-miR-1967 | mmu-miR-743a-3p | mmu-miR-463-3p |
| mmu-miR-486-5p | mmu-miR-1983 | mmu-miR-1193-3p | mmu-miR-875-3p |
| mmu-miR-485-5p | mmu-miR-876-5p | mmu-miR-127-5p | mmu-miR-675-3p |
| mmu-miR-469 | mmu-miR-875-5p | mmu-miR-1196 | mmu-miR-675-5p |
| mmu-miR-153-3p | mmu-miR-767 | mmu-miR-1929-5p | mmu-miR-1940 |
| mmu-miR-193-3p | mmu-miR-671-5p | mmu-miR-700-3p | mmu-miR-689 |
| mmu-miR-670-5p |  |  |  |

**Supplementary Table 1: List of miRNAs downregulated in AA mice.**

| **miRNA upregulated in AA mice** | | **miRNA downregulated in HLSCEV mice** | |
| --- | --- | --- | --- |
| mmu-miR-342-3p | mmu-miR-377-3p | mmu-miR-654-5p | mmu-miR-184-3p |
| mmu-miR-466e-5p | mmu-miR-375-3p | mmu-miR-296-3p | mmu-miR-154-5p |
| mmu-miR-466d-5p | mmu-miR-467a-5p | mmu-miR-465a-3p | mmu-miR-138-5p |
| mmu-miR-466b-3p | mmu-miR-329-3p | mmu-miR-207 | mmu-miR-133b-3p |
| mmu-miR-466a-5p | mmu-miR-294-3p | mmu-miR-409-3p | mmu-miR-101a-3p |
| mmu-miR-466a-3p | mmu-miR-146b-5p | mmu-miR-542-5p | mmu-miR-30b-5p |
| mmu-miR-465c-3p | mmu-miR-21a-5p | mmu-miR-325-3p | mmu-miR-26b-5p |
| mmu-miR-450b-3p | mmu-miR-882 | mmu-miR-329-3p | mmu-miR-302a-3p |
| mmu-miR-467h | mmu-miR-883a-3p | mmu-miR-367-3p | mmu-miR-1b-5p |
| mmu-miR-34b-3p | mmu-miR-1902 | mmu-miR-377-3p | mmu-miR-876-5p |
| mmu-miR-34b-5p | mmu-miR-1961 | mmu-miR-448-3p | mmu-miR-1941-3p |
| mmu-miR-223-3p | mmu-miR-880-3p | mmu-miR-452-5p | mmu-miR-712-5p |
| mmu-miR-31-5p | mmu-miR-9-3p | mmu-miR-466h-5p | mmu-miR-741-3p |
| mmu-miR-466b-3-3p | mmu-miR-10a-3p | mmu-miR-488-3p | mmu-miR-880-3p |
| mmu-miR-34c-5p | mmu-miR-130b-5p | mmu-miR-504-5p | mmu-miR-1197-3p |
| mmu-miR-142a-5p | mmu-miR-136-3p | mmu-miR-376c-3p | mmu-miR-9-3p |
| mmu-miR-190b-5p | mmu-miR-218-2-3p | mmu-miR-302c-3p | mmu-miR-124-5p |
| mmu-miR-146a-5p | mmu-miR-297c-3p | mmu-miR-297c-5p | mmu-miR-203-5p |
| mmu-miR-448-3p | mmu-miR-376c-5p | mmu-miR-294-3p | mmu-miR-297c-3p |
|  |  | mmu-miR-219a-5p | mmu-miR-374b-3p |
|  |  | mmu-miR-208b-3p | mmu-miR-376b-5p |
|  |  | mmu-miR-205-5p | mmu-miR-376c-5p |
|  |  | mmu-miR-201-5p | mmu-miR-433-5p |
|  |  |  | mmu-miR-463-5p |

**Supplementary Table 2: List of miRNAs upregulated in AA mice, and list of miRNAs downregulated in AA mice treated with HLSC-EVs.**

| **miRNA upregulated in HLSC-EV mice** | | |
| --- | --- | --- |
| mmu-miR-669g | mmu-miR-212-3p | mmu-miR-875-3p |
| mmu-miR-188-3p | mmu-miR-208a-3p | mmu-miR-742-5p |
| mmu-miR-466f | mmu-miR-187-3p | mmu-miR-708-3p |
| mmu-miR-291a-5p | mmu-miR-503-5p | mmu-miR-467e-3p |
| mmu-miR-297b-5p | mmu-miR-133a-3p | mmu-miR-382-3p |
| mmu-miR-327 | mmu-miR-669i | mmu-miR-302c-5p |
| mmu-miR-331-5p | mmu-miR-1983 | mmu-miR-300-5p |
| mmu-miR-369-3p | mmu-miR-1958 | mmu-miR-293-5p |
| mmu-miR-449b | mmu-miR-1942 | mmu-miR-127-5p |
| mmu-miR-450b-3p | mmu-miR-1933-5p | mmu-miR-145a-3p |
| mmu-miR-495-3p | mmu-miR-1904 | mmu-miR-669k-3p |
| mmu-miR-490-3p | mmu-miR-876-3p | mmu-miR-1964-3p |
| mmu-miR-469 | mmu-miR-875-5p | mmu-miR-669m-3p |
| mmu-miR-464 | mmu-miR-1950 | mmu-miR-2135 |
| mmu-miR-463-3p | mmu-miR-2183 | mmu-miR-689 |
| mmu-miR-363-3p | mmu-miR-719 | mmu-miR-881-3p |
| mmu-miR-302b-3p | mmu-miR-693-5p | mmu-miR-154-3p |
| mmu-miR-220 | mmu-miR-688 | mmu-miR-743a-3p |

**Supplementary Table 3: List of miRNAs upregulated in AA mice treated with HLSC-EVs**
